# Genetic Variations in Prostaglandin E_2_ Pathway Identified as Susceptibility Biomarkers for Gastric Cancer in an Intermediate Risk European Country

**DOI:** 10.3390/ijms22020648

**Published:** 2021-01-11

**Authors:** Catarina Lopes, Carina Pereira, Mónica Farinha, Rui Medeiros, Mário Dinis-Ribeiro

**Affiliations:** 1 Molecular Oncology and Viral Pathology Group, IPO Porto Research (CI-IPOP), Portuguese Institute of Oncology, Rua Dr. António Bernardino de Almeida, 4200-072 Porto, Portugal; catarina.p.lopes@ipoporto.min-saude.pt (C.L.); ruimedei@ipoporto.min-saude.pt (R.M.); 2 CINTESIS—Center for Health Technology and Services Research, University of Porto, Rua Dr. Plácido da Costa, 4200-450 Porto, Portugal; mdinisribeiro@gmail.com; 3 Pathology Department, Portuguese Institute of Oncology, Rua Dr. António Bernardino de Almeida, 4200-072 Porto, Portugal; monicadfarinha@gmail.com; 4 Portuguese League Against Cancer, Estrada Interior da Circunvalação, 4200-172 Porto, Portugal; 5 Gastroenterology Department, Portuguese Institute of Oncology, Rua Dr. António Bernardino de Almeida, 4200-072 Porto, Portugal

**Keywords:** gastric cancer, genetic susceptibility, prostaglandin E_2_, prostaglandin-endoperoxide synthase 2, hydroxyprostaglandin dehydrogenase 15-(NAD), solute carrier organic anion transporter family member 2A1, ATP binding cassette subfamily C member 4

## Abstract

The cyclooxygenase-2 (COX-2)/prostaglandin E_2_ (PGE_2_) pathway exerts deleterious pleiotropic effects in inflammation-induced gastric carcinogenesis. We aimed to assess the association of genetic variants in prostaglandin-endoperoxide synthase 2 (*PTGS2*), ATP binding cassette subfamily C member 4 (*ABCC4*), hydroxyprostaglandin dehydrogenase 15-(NAD) *(HPGD*), and solute carrier organic anion transporter family member 2A1 (*SLCO2A1*) PGE_2_ pathway-related genes with gastric cancer (GC) risk in a European Caucasian population. A hospital-based case-control study gathering 260 GC cases and 476 cancer-free controls was implemented. Using a tagSNP approach, 51 single nucleotide polymorphisms (SNPs) were genotyped through MassARRAY^®^ iPLEX Gold Technology or allelic discrimination by real-time polymerase chain reaction (PCR). Homozygous carriers of the minor allele for both rs689466 and rs10935090 SNPs were associated with a 2.98 and 4.30-fold increased risk for GC, respectively (95% confidence interval (CI): 1.14–7.74, *p* = 0.027; 95% CI: 1.22–15.16, *p* = 0.026), with the latter also being associated with an anticipated diagnosis age. A multifactor dimensionality reduction analysis identified an overall three-factor best interactive model composed of age, rs689466, and rs1678374 that was associated with a 17.6-fold GC increased risk (95% CI: 11.67–26.48, *p* < 0.0001, (cross-validation) CV consistency of 8/10 and accuracy of 0.807). In this preliminary study, several tagSNPs in PGE_2_ pathway-related genes were identified as risk biomarkers for GC development. This approach may help to identify higher-risk individuals and may contribute to the tailoring screening of GC in intermediate-risk European countries.

## 1. Introduction

The asymptomatic nature of early gastric cancer (GC) and the failure to identify high-risk individuals often result in GC diagnosis in advanced stages of the disease and a consequent poor prognosis [1,2]. In Western countries, such as Portugal, it is suggested that the most effective approach to reduce GC mortality is through the identification of risk factors, allowing personalized screening, diagnosis, and surveillance [3].

Prostaglandin E_2_ (PGE_2_), through its pleiotropic effects, is involved in most hallmarks of cancer, namely evasion of apoptosis, sustained angiogenesis, tissue invasion [4,5,6]. Cyclooxygenase-2 (COX-2) is the rate-limiting enzyme in PGE_2_ synthesis and its role in cancer has been extensively studied in a variety of cancers [7,8,9]. PGE_2_ becomes available to interact with the prostanoid receptors and exert its deleterious effects, being transported out of the cell by multidrug resistance protein 4 (MRP4) [10]. The levels of this lipid are not only determined by its synthesis but also by the degradation rates, which depend on internalization and inactivation [10]. Once PGE_2_ is transported back into the cell via prostaglandin transporter (PGT), it is then inactivated by 15-hydroxyprostaglandin dehydrogenase (15-PGDH) [11].

The most comprehensive review study on the association between genetic variations and GC risk is the field synopsis and meta-analysis by Mocellin and colleagues published in 2015 [12]. Overall, they found eleven polymorphisms significantly associated with risk to develop the disease in the *MUC1, MTX1, PSCA, PRKAA1, PLCE1, TGFBR2, PKLR, GSTP1, CASP8,* and *TNF* genes [12]. It is noteworthy that the highest percentage of polymorphisms significantly associated with GC risk belonged to the immunity/inflammation group (36%), where *PTGS2* was included [12]. Additionally, over one hundred variants with lower quality significant associations were also identified [12].

We recently reported a dysregulation of the PGE_2_ pathway in GC in a Caucasian population [13]. Moreover, genetic variants in the genes encoding the four proteins mentioned above (*PTGS2, ABCC4, SLCO2A1*, and *HPGD*, respectively) have been previously characterized in our population in colorectal cancer susceptibility and clinical outcome [14,15,16]. Nevertheless, the characterization of these four genes in GC is rather scarce and most of the published studies focus on Asian patients, even though differences across ethnicities are expected [17,18,19].

Thus, the main purpose of this study was to characterize the genomic profile of the PGE_2_ pathway associated with GC development in a Caucasian population by targeting the *PTGS2, ABCC4, HPGD,* and *SLCO2A1* genes using a tagSNP approach Additionally, we further aimed to evaluate the influence of the addressed genetic polymorphisms on the mRNA expression of those genes. The identification of higher-risk individuals to targeted early screening and diagnosis could prove to be a valuable and cost-effective approach towards GC mortality reduction in low to intermediate risk countries, such as Portugal, where mass screening is unwarranted.

## 2. Results

### 2.1. Study Population

The characterization of the study population is summarized in Table 1. Cases were significantly older than controls (median age of 70 vs. 58, respectively, *p* < 0.001), whereas no differences were noticeable in male distribution (58% and 66% in cases and controls, respectively, *p* = 0.057). Most tumors were located in the antrum and corpus-antrum transition (62%) and described as moderately differentiated (61%). Regarding tumor staging, nearly 60% of GC patients were diagnosed in stages I and II (56%).

### 2.2. Genotype Frequencies and Risk Estimates

Selected SNPs are summarized in Supplementary Table S1. Nine SNPs were excluded, five due to genotyping failure and the other four due to deviation from HWE (*p* < 0.05). Thus, a total of 51 SNPs were included in this analysis. The concordance rates were 100% for all genetic polymorphisms and the mean genotype call rate was 99.9%. Overall, 8 genetic polymorphisms were implicated in the susceptibility for GC development, as displayed in Table 2. The risk estimates for the involvement of all analyzed genetic variants in GC onset are summarized in Supplementary Table S2.

Homozygous individuals for the minor G allele of the rs689466 polymorphism in the *PTGS2* gene were overrepresented in the GC patient group, leading to a three-fold increase of GC risk (aOR = 2.98; 95% CI: 1.14–7.74; *p* = 0.027) in the multivariate analysis, including age and sex as covariates. Moreover, the Kaplan-Meier analysis showed that the estimated age at diagnosis is three years anticipated for these individuals when compared with the ones carrying the A allele (70 vs. 73 years; 95% CI: 71.48–74.52 and 62.49–77.52, respectively; *p* = 0.011).

Following a recessive model, the rs1678374 and rs1678405 polymorphisms in the *ABCC4* gene were associated with a 51% protection for GC development in homozygous carrying the C allele (aOR = 0.49; 95% CI: 0.26–0.91; *p* = 0.019 and aOR = 0.49; 95% CI: 0.23–1.03; *p* = 0.049, respectively). Additionally, for the rs1751031 polymorphism in the same gene and following a dominant model, carriers of the minor allele were also associated with a 40% protection (aOR = 0.60; 95% CI: 0.39–0.94; *p* = 0.022).

Regarding the *HPGD* gene, the rs2303520 polymorphism was associated with a 65% increased risk for GC onset for carriers of GA genotype compared to homozygous individuals, following the overdominant model of inheritance (aOR = 1.65; 95% CI: 1.05–2.59; *p* = 0.031).

Three SNPs in the *SLCO2A1* gene showed an influence in GC susceptibility. Carriers of the rs11915399T allele presented a 38% decreased risk for this type of cancer (OR = 0.62; 95% CI: 0.39–0.99; *p* = 0.043), whereas both the rs10935090 and rs9821091 tagSNPs were associated with an increased risk in individuals carrying the homozygous minor allele genotype, with the former reaching over four-fold enhanced susceptibility (OR = 4.30; 95% CI: 1.22–2.53; *p* = 0.026 and OR = 1.95; 95% CI: 1.12–3.40; *p* = 0.02, respectively). Furthermore, carriers of the TT genotype in rs10935090 genetic variation showed a statistically significant ten-year anticipation in the estimated age at diagnosis compared to individuals carrying the C allele (62 vs. 72 years; 95% CI: 59.61–64.39 and 71.81–74.19, respectively; *p* < 0.001).

Despite not being identified as susceptibility biomarkers for GC development, the time-to-diagnosis analysis showed that the rs2555632 tagSNP in the *HPGD* gene and the rs4241362 genetic polymorphism in the *SLCO2A1* gene were linked to anticipation in the age of diagnosis by two and seven years, respectively, in CC homozygous individuals compared to carriers of the T allele, as displayed in Supplementary Table S2 (70 vs. 72 years; 95% CI: 66.67–73.33 and 70.73–73.27, respectively; *p* = 0.027, and 65 vs. 72 years; 95% CI: 62.94–67.06 and 70.87–73.13; *p* = 0.024, respectively).

### 2.3. Functional Characterization of the GC-Associated Biomarkers

**Figure 1:**
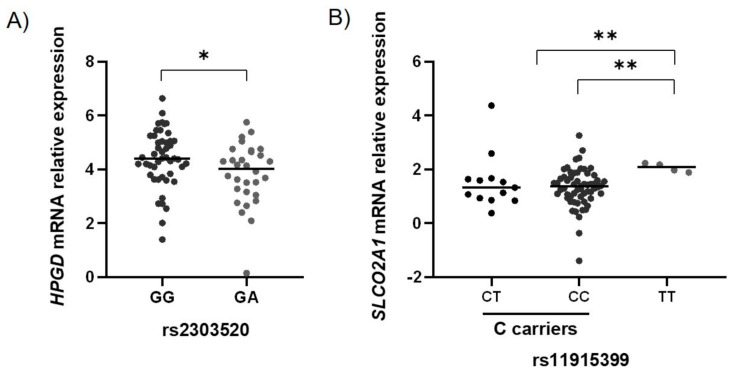
(**A**) Hydroxyprostaglandin dehydrogenase 15-(NAD) (*HPGD*) mRNA relative expression considering the genotypes of the rs2303520 G>A polymorphism. In “normal”-appearing mucosa, the GA genotype is associated with *HPGD* mRNA downregulation by a mean factor of 0.67. Lines represent median values of expression. (**B**) Solute carrier organic anion transporter family member 2A1 (*SLCO2A1*) mRNA relative expression considering the genotypes of the rs11915399 C > T polymorphism. In “normal”-appearing mucosa, the TT genotype is associated with *SLCO2A1* mRNA upregulation compared to the CC genotype and the carriers of the C allele by a mean factor of 1.63 and 1.67, respectively. Lines represent median values of expression. * *p* < 0.05; ** *p* < 0.01.

We observed that the rs230520 and rs11915399 tagSNPs modulated the expression of *HPGD* and *SLCO2A1* genes, respectively. As can be observed in Figure 1A, the GA genotype is associated with a decrease in *HPGD* mRNA expression by a mean factor of 0.67 compared to the heterozygous genotype (4.40 ± 0.16 vs. 3.82 ± 0.21, *p* = 0.027) in “normal”-appearing mucosa samples.

Regarding the rs11915399 tagSNP in the *SLCO2A1* gene, represented in Figure 1B, we noticed an increase in mRNA expression in the minor T allele homozygous individuals compared to both the carriers of the major C allele (2.07 ± 0.83 vs. 1.36 ± 0.09, *p* = 0.007) and the CC genotype alone (1.32 ± 0.09, *p* = 0.006) by a mean factor of 1.67 and 1.63, respectively.

### 2.4. Haplotype Analysis

Since multiple SNPs were highlighted within *ABCC4* and *SLCO2A1* genes, a haplotype analysis was performed, and the derived haplotypes frequencies are displayed in Table 3. The most frequent one (TTA) was present in 42% of controls and was used as the reference haplotype in the *ABCC4* gene. Only the block containing the rs1678374C, rs1678405C, and rs1751031G alleles, CCG, showed an influence in GC susceptibility, presenting a 53% protection for GC onset, which is consistent with the individual SNP analysis (aOR = 0.47; 95% CI: 0.23–0.93; *p* = 0.032).

The reference haplotype for the *SLCO2A1* gene, CCG, was present in over 45% of controls. A 2.8-fold increased risk was observed for individuals carrying the block TCA (95% CI: 1.41–5.48; *p* = 0.0034), which contains the alleles associated with increased risk in the single analysis of the rs10935090 and rs9821091 genetic polymorphisms (minor T allele and minor A allele, respectively). The rs11915399C allele is also included in that block, which is coherent with the association reported between its opposing rs11925399T allele and GC protection.

### 2.5. Gene-“Environment” Interaction Analysis

An MDR approach was carried out to assess possible interactions between the most meaningful SNPs, i.e., the polymorphisms associated with GC onset in the main and the best interactive models are summarized in Table 4. We further included the age and sex variables. All the addressed models were significant at *p* < 0.0001 and the highest CVC was observed for the model with one single factor (10/10). Nevertheless, the best three-factor interaction model, which included age, *PTGS2* rs689466 and *ABCC4* rs1678374 SNPs, presented the highest testing accuracy of 81% with a CVC of 8/10. This gene-gene interaction was associated with a 17.6-fold increase in GC risk.

Upon performing the FDR analysis to address multiple testing correction, none of the genetic biomarkers previously associated with GC susceptibility retained its statistical significance.

## 3. Discussion

The pleiotropic activities of the PGE_2_/COX-2 pathway and their effect on cancer progression have been reviewed and explored throughout the years [5,20,21,22]. Dysregulation of this pathway due to COX-2/MRP4 overexpression and PGT/15-PGDH downregulation has been shown to lead to the accumulation of PGE_2_ in the extracellular microenvironment and, therefore, to contribute to its nefarious effects in gastrointestinal models [5,23].

Differences in the genetic and molecular signatures across ethnic populations have also been reported [17,18,19,24]. Nevertheless, most of the published studies exploring GC focused on Asian populations, and thus the role of the PGE_2_ pathway remains elusive in Western countries. Our group has previously explored the involvement of genetic variants in this pathway in colorectal cancer development and has recently reported its dysregulation in GC in a Caucasian population [13,14]. In this study, we hypothesized that targeting the genetic variability in *PTGS2*, *ABCC4*, *HPGD,* and *SLCO2A1*, main players in the PGE_2_ pathway, would enable the identification of susceptibility biomarkers for GC onset. Considering this, a hospital-based case-control study was implemented in the major Oncology Institute of the Northern region of Portugal.

In this preliminary study, eight genetic polymorphisms were observed to influence the risk of GC development. We noticed an association between the minor rs689466G allele and a 3-fold increased risk for GC onset. It is known that this SNP is located in the *PTGS2* promoter region at −1195 bp from the transcription start site, which is rich in several *cis*-regulatory elements involved in *PTGS2* transcription [25]. Although we did not observe a repercussion of this SNP in mRNA expression, we have to mention a limited number of mRNA expression results for this gene. Furthermore, the presence of the G allele has been previously associated with higher transcriptional activity of *PTGS2* in colorectal cell lines and the implication of this SNP in colorectal cancer susceptibility had already been reported by our group and in other Caucasian populations [26,27]. This result contradicts the reports of a meta-analysis by Luo et al. [17], which identifies the rs689466A allele as an increased risk genetic biomarker for GC. Additionally, the rs689466A allele has also been shown to create a binding-site for a transcription-factor, c-MYB, which enhances *PTGS2* transcriptional activity. Also, another meta-analysis by Wang et al. [28] reports an association between COX-2 rs689466A allele and increased risk of several cancers, including hepatocellular carcinoma, pancreatic, and gastric cancer. Nevertheless, it should be noted that the studies included in these meta-analyses were performed almost exclusively in Asian populations. Moreover, a study by Zamudio et al. [29] revealed different allele frequencies for this SNP between populations with distinct ancestry and the authors argue the need to control continental ancestry when performing association studies on gastric cancer.

In the *ABCC4* gene, the largest gene under study, the rs1678374, rs1678405, and rs1751031 polymorphisms were found to be associated with GC susceptibility. A 50% protection for GC onset was observed for rs1678374CC genotype carriers. Although no previous reports have been published suggesting an influence of this SNP in disease development, it is known to be located within an intron (intron 9). Even though this genetic variation is not expected to affect the amino acid sequence, genetic variation in noncoding DNA sequences, such as introns, can have important functional and regulatory roles [30,31]. On the other hand, our previous findings in colorectal cancer support the decreased risk associations here highlighted for the rs1678405C allele and AG genotype of the rs1751031 SNP [14,16]. These two polymorphisms belong to the same LD block and are also located in introns.

In this study, we observed that the rs2303520GC heterozygous genotype was not only associated with a 65% GC increased risk, but also with *HPGD* mRNA decreased expression. The underexpression of *HPGD* mRNA is expected to impair PGE_2_ inactivation within the cell, leading to an increase in PGE_2_ concentration and, consequently, to its nefarious effects in the extracellular milieu, thus supporting the observed increased risk for GC.

Regarding the gene encoding the PGT protein, the *SLCO2A1*, we reported a four and two-fold increased risk for GC onset in our population for the rs10935090TT and the rs9821091AA genotypes, respectively. The former is known to be located in exon 1 and to represent a synonymous variant and the latter to be located in an intron [30]. On the other hand, carriers of the rs11915399T allele exhibited a decreased risk for GC, followed by an increased *SLCO2A1* mRNA expression compared to C allele and CC genotype carriers, contributing to PGE_2_ transport back into the cell by PGT and, consequently, its inactivation and a decrease in GC risk.

In complex diseases, the epistasis analysis may help understand a likely source of heritability, allowing the definition of genetic and gene-environment signatures for the development of GC, which may represent an important key for GC prevention and early detection [32]. Furthermore, it might also provide some insights into how these genes are regulated and regulate each other. In this study, we observed that the models with the highest CV accuracy included three factors, although not presenting the best CVC (8/10). Overall, the best three-factor model, including age, the rs689466 and rs1678374 SNPs, revealed an 81% CV accuracy and was associated with a 17.6-fold increased risk for GC development.

A major limitation inherent to our study is the multiple testing problem, which we corrected using FDR, but, due to our restricted statistical power, none of the SNPs we found to be associated with GC retained their statistical significance. To overcome that, we would need to increase the number of samples in future studies, so we can increase the statistical power and, thus, the precision of our results. Our control population was represented by unscreened blood donors. Eighty-five percent of these participants were asymptomatic and still blood donors five to seven years after recruitment, and the remaining subjects were excluded from blood donation due to age criteria, with no records of gastrointestinal malignancy. Thus, it is highly unlikely that crossover had occurred. Also, due to the low recruitment rate, we had to resort to FFPE samples archived at the Pathology department at IPO-Porto to characterize our group of GC patients. We have previously validated the use of these tissues for genotype characterization in colorectal cancer patients and our concordance rate between replicates was perfect [14]. Nowadays, PCR techniques allow the use of FFPE tissue samples, but they require optimization, short amplicons, and rely on the quantity and quality of the extracted RNA [33,34]. The selected amplicons for this study ranged between 61 and 88 bp, the mean optical density (OD) was 1.95 for normal and 1.96 for tumor samples, and 20 ng of cDNA was used, well above the lower sensitivity limit. It is also known that C_T_ values increase with RNA degradation, which occurs during the tissue fixation and embedding process [35]. FFPE samples, particularly, present a high degree of RNA degradation, which could affect accuracy and sensitivity of the real-time PCR quantification [35]. Nevertheless, many genes can be reliably measured and Walter et al. [36] even described successful and reproducible RT and PCR amplification for FFPE samples, showing no inhibitory effect of the formalin. Even though we did not match controls to cases, the effect of potential confounding variables in the statistical analysis, such as age, was minimized through multivariate analysis. Moreover, future studies should include, whenever possible, information concerning other important risk factors, such as *Helicobacter pylori* infection, smoking, diet, and obesity. For the genomic characterization of the PGE_2_ pathway, we applied a tagSNP approach, which allowed us to capture the majority of SNP variation in a genome region, reducing the necessity of a large amount of sample and the genotyping costs. The tagSNPs transferability in distinct populations has been explored in several studies [37,38,39,40]. Our panel was retrieved from the CEU population, represented by Utah residents with Northern and Western European ancestry, of the International HapMap project [41]. It is noteworthy that this is one of the first studies to be performed in a Caucasian population and a country with intermediate GC risk.

Currently, in Western countries, mass screening of GC is unwarranted [3]. Therefore, targeted early screening and diagnosis of higher-risk individuals might prove to be cost-effective in low to intermediate risk European countries, such as Portugal.

## 4. Materials and Methods

### 4.1. Study Population

This non-matched hospital-based case-control study was approved by the Ethics Committee at the Instituto Português de Oncologia do Porto (IPO-Porto) on 15th of December 2016 (CES.314/016) and included 736 participants: 476 cancer-free controls and 260 histologically confirmed intestinal-type GC patients. All the individuals were from the Northern region of Portugal and recruited at IPO-Porto.

In the control group, individuals without clinical evidence of GC or any other malignancy with or above 50 years old were included. They were randomly recruited from the service of blood donation at IPO-Porto between July 2005 and February 2008 and integrated a DNA database of over one thousand blood donors. All controls complying with the age inclusion criteria have been previously characterized [14].

The GC patients group gathered patients with age equal or superior to 50 years old with histological confirmation of intestinal-type GC between May 2012 and December 2015. These patients were consecutively selected after reviewing the histopathological database from the Pathology department at IPO-Porto, based on the availability of formalin-fixed paraffin-embedded (FFPE) samples.

Medical records were reviewed to extract the clinicopathological variables, such as localization, stage, and tumor grade. All tumors were restaged according to the eighth edition of the AJCC Cancer Staging Manual [42].

### 4.2. Sample Collection and Processing

The nucleic acids were extracted from up to 6 cm^2^ of macrodissected FFPE areas, enriched in “normal” or tumor cells, using the AllPrep^®^ DNA/RNA FFPE Kit (Qiagen, Hilden, Germany) following the manufacturer instructions and quantified by NanoDrop^TM^ Lite Spectrophotometer (Thermo Fisher Scientific, Waltham, MA, USA). The quality was assessed by measuring the optical density (OD) 260/280 ratio and samples were kept at −20 °C until further processing.

### 4.3. Genetic Polymorphisms Selection and Characterization

The method for polymorphism selection has been previously described^14^. Briefly, 55 tagSNPs were included after being retrieved from a set of common SNPs in the Caucasian population of the HapMap project (CEU): (1) with a minor allele frequency equal or superior to 0.15; (2) within the coding region of the gene plus 2 kb upstream and downstream; (3) with an r^2^ superior to 0.8 and (4) that successfully converted to the Sequenom platform.

The tagSNP genotyping was performed using MassARRAY^®^ iPLEX Gold Technology (Agena Bioscience, San Diego, CA, USA) based on multiplex amplification followed by mass-spectrometric product separation. This method was carried out by the Centro Nacional de Genotipado at the Universidade de Santiago de Compostela, Spain. A total of 250 samples were sent with DNA concentration ≥20 ng/µL. Of those, 241 samples were considered of good quality and successfully genotyped.

Furthermore, the rs5275, rs689466, and rs20417 polymorphisms in *PTGS2* gene and the rs2555639 and rs2612656 polymorphisms in *HPGD* gene, previously associated with tumor development [28,43,44,45,46], were characterized in 198 GC patients with available DNA sample, through allelic discrimination using the validated TaqMan^®^ SNP genotyping assays (Thermo Fisher Scientific, Waltham, MA, USA) C__7550203_10, C__2517145_20, C__11997909_40, C__16038735_10, and C__15909858_20 respectively, by Real-Time PCR.

Genotypes were excluded from the analysis if the following was observed: call rate inferior to 0.90, concordance rate inferior to 0.95, or Hardy–Weinberg equilibrium (HWE) with *p* < 0.05 in the control population. Ten percent of the samples were resubmitted to a new genetic characterization by random selection to confirm the results.

### 4.4. Reverse Transcription Reaction

Approximately one hundred samples of “normal”-appearing mucosa and tumoral mucosa of GC patients were randomly selected.

Complementary DNA (cDNA) was synthesized from up to 2 µg of RNA using the High-Capacity cDNA Reverse Transcription (RT) kit (Thermo Fisher Scientific, Waltham, MA, USA) following the manufacturer’s instructions.

All RT reactions included one no-template negative control. Moreover, 1 µL containing 1 µg of the QPCR Human Reference Total RNA, part of the Absolutely RNA FFPE Kit (Agilent, Santa Clara, CA, USA), was used as a positive control to monitor the quality of the RT.

### 4.5. Real-Time PCR

cDNA amplification by Real-Time PCR was performed using a StepOne Plus Real-Time PCR system (Applied Biosystems, Foster City, CA, USA). In a 10 µL reaction mix, 5.0 µL of TaqMan^®^ Gene Expression Master Mix (Applied Biosystems, Foster City, CA, USA), 0.5 µL of TaqMan^®^ Gene Expression Assay (Applied Biosystems, Foster City, CA, USA), and 20 ng of cDNA template were used.

The gene expression assays used to measure the mRNA expression of the *PTGS2*, *HPGD*, *ABCC4*, and *SLCO2A1* genes were Hs00153133_m1, Hs00168359_m1, Hs00988717_m1, and Hs01114926_m1 (Applied Biosystems, Foster City, CA, USA), respectively. All assays underwent the following thermal cycling conditions: 50 °C for 2 min, 95 °C for 10 min, and 45 cycles of 95 °C for 15 s and 60 °C for 1 min. They were validated to determine the efficiency of the amplification reaction and their limit of detection using a 1:2 dilution series with 7 dilution steps. Efficiency between 90% and 105% and sensitivity above 6.25 ng were reported for all gene expression assays used.

For mRNA quantification using real-time PCR, triplicates were used and replicates with a standard deviation (SD) superior to 30% of 1 C_T_ were excluded. For each sample, all target genes and reference ones were amplified on the same plate. One positive control from the RT reaction and three no template negative controls were included. The endpoint of the real-time PCR was the cycle threshold (C_T_) determined as the average value from three independent reactions. The best reference gene pair to be used in gene expression normalization, *HPRT1* and *IPO8* (gene expression assays Hs02800695_m1 and Hs00914057_m1, respectively, Applied Biosystems, Foster City, CA, USA), was chosen after identifying the most stable, from a panel of six genes (*B2M*, *HPRT1, RPL29, PPIA, IPO8,* and *GUSB*) retrieved after reviewing the literature on gastrointestinal cancers [47,48] and using the NormFinder and geNorm software. A smaller percentage of mRNA positive samples were observed for the *PTGS2* gene (56.6% in normal and 70.8% in tumor samples) compared to *ABCC4, HPGD,* and *SLCO2A1* genes (100%, 100% and 99.0% in normal tissues vs. 100%, 93.8%, and 90.6% in tumor samples, respectively).

### 4.6. Statistical Analysis

Data analysis was performed using the computer software IBM^®^ Statistical Package for the Social Sciences (SPSS^®^) Statistics (IBM Corp., Armonk, NY, USA) version 26.0 for Windows. Categorical variables were compared using chi-square (Χ^2^) analysis with a 5% level of significance and nonparametric (Mann-Whitney) tests were used to compare mean values. The Hardy–Weinberg Equilibrium (HWE) was tested by the Pearson’s goodness of fit test to compare the genotype frequencies observed versus the expected. If the *p* value was inferior to 0.05, control genotype distributions were assumed to deviate from HWE.

Odds ratio (OR) and its confidence interval (CI) were estimated by a multivariate logistic regression analysis as a measure of association between the polymorphisms and the risk for GC development. Age and sex were included as covariates in this analysis and homozygotes for the allele with the highest frequency were defined as the reference genotype for OR estimation. We tested the following models of inheritance: codominant, dominant, recessive, and overdominant.

For the haplotype analysis performed at a gene level, the implementation of the expectation-maximization (EM) algorithm coded into the haplo.stats package was used to estimate haplotype frequencies. The reference group was automatically selected and corresponded to the most frequent haplotype and the haplotype blocks were defined considering the most meaningful SNPs. Kaplan–Meier analysis was performed using a log-rank statistical test to assess the correlation between the genetic polymorphisms and the age at diagnosis.

The “gene-environment” interactions in GC development were assessed by applying a nonparametric approach using the open-source multifactor dimensionality reduction (MDR) software (version 3.0.2) (https://www.multifactordimensionalityreduction.org/). The competence of an MDR model is evaluated by its testing accuracy and cross-validation consistency (CVC) and, in general, the single best one reaches the maximum of these two parameters.

The false discovery rate (FDR) was used to correct for multiple testing and confirm the noteworthiness of significant findings [49,50]. This was performed using the SPSS software.

The relative mRNA expression was expressed as the difference between C_T_s correspondent to the amplification curves of the target genes (*PTGS2, ABCC4, HPGD,* and *SLCO2A1*) and the reference genes (-∆C_T_). The expression fold-change was calculated following the Livak method (2^−∆∆Ct^) [51]. To determine if the genetic variants identified as susceptibility biomarkers for GC in this study could be determinants of mRNA expression, we performed one-way ANOVA to compare the mean tissue expression between the three possible genotypes and student’s t or nonparametric tests when appropriate to assess the mean tissue expression between two genotypes or models of inheritance. GraphPad Prism version 8.00 for Windows was used to obtain graphical designs.

## Figures and Tables

**Table 1:** Description of participants.

		Cases (n = 260)	Controls (n = 476)	*p* Value
**Demographics**				
Age (years)				
Mean ± SD		69.87 ± 0.60	57.98 ± 0.23	< 0.001
Median (min-max)		70 (50–92)	58 (50–69)	
Sex, n (%)				
Male		152 (58.5)	312 (65.5)	0.057
Female		108 (41.5)	164 (34.5)	
**Tumor characteristics**				
Tumor location, n (%)				
Cardia and GEJ		24 (9.4)	--	
Fundus and corpus		41 (16.1)	--	
Antrum and corpus-antrum transition		157 (61.6)	--	
Angularis incisura		7 (2.7)		
Others *		26 (10.2)		
Grade, n (%)				
Well-differentiated		28 (10.8)	--	
Moderately differentiated		157 (60.6)	--	
Poorly differentiated		63 (24.3)	--	
Cannot be assessed		11 (4.2)	--	
Stage, n (%)				
I-II		145 (56.0)	--	
III-IV		114 (44.0)	--	
Synchronous tumors, n (%)				
Yes		6 (2.3)	--	
No		254 (97.7)	--	

SD: standard deviation; GEJ: gastroesophageal junction. * Including tumors that occupy more than one location and tumors of the gastric stump. For synchronous tumors, the most advanced lesion was considered in the characterization.

**Table 2:** Genotype frequencies among gastric cancer cases and controls, risk estimates for the involvement of *PTGS2, ABCC4, HPGD, and SLCO2A1* genetic variants in gastric cancer onset and estimated age at diagnosis.

SNP	Model	Genotype Frequencies		Univariate Analysis			Multivariate Analysis			Age at Diagnosis		
		Cases, *n* (%)	Controls, *n* (%)	OR	95% CI	*p* Value	aOR	95% CI	*p* Value	Median (years)	95% CI	*p* Value
***PTGS2***												
rs689466	Codominant											
	AA	121 (61.1)	322 (68.8)	1.00	-	0.054	1.00	-	**0.021**	73.00	71.25–74.75	-
	AG	63 (31.8)	130 (27.8)	1.29	0.89–1.86		1.50	0.93–2.42		73.00	68.89–77.11	0.21
	GG	14 (7.1)	16 (3.4)	**2.33**	**1.10–4.92**		**3.40**	**1.29–8.97**		**70.00**	**62.49–77.52**	**0.008**
	Dominant											
	AA	121 (61.1)	322 (68.8)	1.00	-	0.056	1.00	-	**0.022**	73.00	71.25–74.75	-
	AG-GG	77 (38.9)	146 (31.2)	1.40	0.99–1.98		**1.69**	**1.08–2.65**		73.00	69.94–76.06	0.058
	Recessive											
	AA-AG	184 (92.9)	452 (96.6)	1.00	-	**0.046**	1.00	-	**0.027**	73.00	71.48–74.52	-
	GG	14 (7.1)	16 (3.4)	**2.15**	**1.03–4.49**		**2.98**	**1.14–7.74**		**70.00**	**62.49–77.52**	**0.011**
	Overdominant											
	AA-GG	135 (68.2)	338 (72.2)	1.00	-	0.30	1.00	-	0.19	73.00	71.46–74.54	-
	AG	63 (31.8)	130 (27.8)	1.21	0.85–1.74		1.37	0.86–2.19		73.00	68.89–77.11	0.38
	Log-additive	-		**1.40**	**1.06–1.86**	**0.021**	**1.66**	**1.15–2.40**	**0.007**	-		
***ABCC4***												
rs1678374	Codominant											
	TT	90 (40.5)	161 (33.9)	1.00	-	0.076	1.00	-	0.063	72.00	69.48–74.52	-
	TC	107 (48.2)	234 (49.3)	0.82	0.58–1.15		1.04	0.67–1.63		72.00	69.74–74.26	0.47
	CC	25 (11.3)	80 (16.8)	**0.56**	**0.33–0.94**		**0.50**	**0.26–0.97**		73.00	71.00–75.00	0.57
	Dominant											
	TT	90 (40.5)	161 (33.9)	1.00	-	0.09	1.00	-	0.55	72.00	69.48–74.52	-
	TC-CC	132 (59.5)	314 (66.1)	0.75	0.54–1.04		0.88	0.58–1.34		72.00	70.77–73.23	0.66
rs1678374	Recessive											
	TT-TC	197 (88.7)	395 (83.2)	1.00	-	0.05	1.00	-	**0.019**	72.00	70.38–73.63	-
	CC	25 (11.3)	80 (16.8)	0.63	0.39–1.01		**0.49**	**0.26–0.91**		73.00	71.00–75.00	0.39
	Overdominant											
	TT-CC	115 (51.8)	241 (50.7)	1.00	-	0.79	1.00	-	0.28	72.00	70.46–73.54	-
	TC	107 (48.2)	234 (49.3)	0.96	0.70–1.32		1.25	0.83–1.89		72.00	69.74–74.26	0.31
	Log-additive	-		**0.77**	**0.60–0.97**	**0.027**	0.78	0.58–1.06	0.11	-		
rs1678405	Codominant											
	TT	108 (50.2)	196 (41.2)	1.00	-	0.052	1.00	-	**0.09**	72.00	70.40–73.60	-
	TC	91 (42.3)	226 (47.5)	0.73	0.52–1.02		0.81	0.52–1.25		72.00	69.60–74.40	0.58
	CC	16 (7.4)	54 (11.3)	**0.54**	**0.29–0.99**		**0.44**	**0.20–0.95**		74.00	67.60–80.40	0.68
	Dominant											
	TT	108 (50.2)	196 (41.2)	1.00	-	**0.027**	1.00	-	0.13	72.00	70.40–73.60	-
	TC-CC	107 (49.8)	280 (58.8)	**0.69**	**0.50–0.96**		0.73	0.48–1.10		72.00	70.18–73.82	0.70
	Recessive											
	TT-TC	199 (92.6)	422 (88.7)	1.00	-	0.11	1.00	-	**0.049**	72.00	70.58–73.42	-
	CC	16 (7.4)	54 (11.3)	0.63	0.35–1.13		**0.49**	**0.23–1.03**		74.00	67.60–80.40	0.58
	Overdominant											
	TT-CC	124 (57.7)	250 (52.5)	1.00	-	0.21	1.00	-	0.76	72.00	70.46–73.54	-
	TC	91 (42.3)	226 (47.5)	0.81	0.59–1.12		0.94	0.62–1.42		72.00	69.60–74.40	0.48
	Log-additive	-		**0.73**	**0.57–0.94**	**0.015**	**0.72**	**0.52–0.99**	**0.041**	**-**		
rs1751031	Codominant											
	AA	154 (69.4)	296 (62.3)	1.00	-	0.10	1.00	-	0.073	72.00	70.25–73.75	-
	AG	59 (26.6)	164 (34.5)	**0.69**	**0.48**		**0.61**	**0.39–0.95**		72.00	69.72–74.28	0.66
	GG	9 (4.0)	15 (302)	1.15	0.49–2.70		0.57	0.17–1.92		78.00	69.81–86.19	0.29
rs1751031	Dominant											
	AA	154 (69.4)	296 (62.3)	1.00	-	0.068	1.00	-	**0.022**	72.00	70.25–73.75	**-**
	AG-GG	68 (30.6)	179 (37.7)	0.73	0.52–1.03		**0.60**	**0.39–0.94**		72.00	70.08–73.93	0.46
	Recessive											
	AA-AG	213 (96.0)	460 (96.8)	1.00	-	0.55	1.00	-	0.52	72.00	70.61–73.39	-
	GG	9 (4.0)	15 (3.2)	1.30	0.56–3.01		0.68	0.21–2.24		78.00	69.81–86.19	0.31
	Overdominant											
	AA-GG	163 (73.4)	311 (65.5)	1.00	-	**0.034**	1.00	-	**0.036**	72.00	70.28-73.72	**-**
	AG	59 (26.6)	164 (34.5)	**0.69**	**0.48-0.98**		**0.62**	**0.40-0.98**		72.00	69.72-74.28	0.79
	Log-additive	-		0.81	0.61-1.09	0.17	**0.65**	**0.44-0.96**	**0.028**	**-**		
***HPGD***												
rs2303520	Codominant											
	GG	143 (64.4)	339 (71.4)	1.00	-	**0.037**	1.00	-	0.065	72.00	70.89–73.11	-
	GA	76 (34.2)	122 (25.7)	**1.48**	**1.04–2.09**		**1.61**	**1.02–2.54**		72.00	69.12–74.88	0.83
	AA	3 (1.4)	14 (3.0)	0.51	0.14–1.79		0.51	0.11–2.34		69.00	64.20–73.80	0.61
	Dominant											
	GG	143 (64.4)	339 (71.4)	1.00	-	0.066	1.00	-	0.086	72.00	70.89–73.11	-
	GA-AA	79 (35.6)	136 (28.6)	1.38	0.98–1.93		1.47	0.95–2.29		72.00	69.05–74.96	0.92
	Recessive											
	GG-GA	219 (98.7)	461 (97.0)	1.00	-	0.18	1.00	-	0.26	72.00	70.63–73.37	-
	AA	3 (1.4)	14 (3.0)	0.45	0.13–1.59		0.45	0.10–2.04		69.00	64.20–73.80	0.61
	Overdominant											
	GG-AA	146 (65.8)	353 (74.3)	1.00	-	**0.021**	1.00	-	**0.031**	72.00	70.87–73.13	-
	GA	76 (34.2)	122 (25.7)	**1.51**	**1.07–2.13**		**1.65**	**1.05–2.59**		72.00	69.12–74.88	0.81
	Log-additive	-		1.21	0.90–1.64	0.21	1.26	0.86–1.84	0.24	-		
***SLCO2A1***												
rs10935090	Codominant											
	CC	162 (73.0)	378 (79.6)	1.00	-	0.13	1.00	-	**0.026**	73.00	71.81–74.19	-
	CT	54 (24.3)	90 (18.9)	1.40	0.95–2.06	0.13	1.46	0.90–2.39	**0.026**	**70.00**	**67.56–72.44**	**0.034**
	TT	6 (2.7)	7 (1.5)	2.00	0.66–6.04		**4.68**	**1.32–16.6**		**62.00**	**59.61–64.39**	**<0.001**
	Dominant											
	CC	162 (73.0)	378 (79.6)	1.00	-	0.054	1.00	-	**0.038**	73.00	71.81–74.19	-
	CT-TT	60 (27.0)	97 (20.4)	1.44	1.00–2.09		**1.65**	**1.03–2.63**		**70.00**	**67.93–72.07**	**0.007**
	Recessive											
	CC-CT	216 (97.3)	468 (98.5)	1.00	-	0.28	1.00	-	**0.026**	72.00	70.87–73.14	-
	TT	6 (2.7)	7 (1.5)	1.86	0.62–5.59		**4.30**	**1.22–15.2**		**62.00**	**59.61–64.39**	**<0.001**
	Overdominant											
	CC-TT	168 (75.7)	385 (81.0)	1.00	-	0.11	1.00	-	0.19	73.00	71.81–74.19	-
	CT	54 (24.3)	90 (18.9)	1.37	0.94–2.02		1.39	0.86–2.27		70.00	67.56–72.44	0.057
	Log-additive	-		**1.40**	**1.01–1.95**	**0.044**	**1.69**	**1.12–2.53**	**0.012**	-		
rs11915399	Codominant											
	CC	159 (71.6)	326 (68.6)	1.00	-	0.72	1.00	-	0.12	72.00	70.27–73.73	-
	CT	57 (25.7)	135 (28.4)	0.87	0.60–1.24		**0.61**	**0.38–0.99**		73.00	71.47–74.53	0.13
	TT	6 (2.7)	14 (3.0)	0.88	0.33–2.33		**0.75**	**0.22–2.63**		74.00	59.93–88.07	0.52
	Dominant											
	CC	159 (71.6)	326 (68.6)	1.00	-	0.42	1.00	-	**0.043**	72.00	70.27–73.73	-
	CT-TT	63 (28.4)	149 (31.4)	0.87	0.61–1.23		**0.62**	**0.39–0.99**		73.00	71.53–74.47	0.11
rs11915399	Recessive											
	CC-CT	216 (97.3)	461 (97.0)	1.00	-	0.86	1.00	-	0.81	72.00	70.64–73.37	-
	TT	6 (2.7)	14 (3.0)	0.91	0.35–2.41		0.86	0.25–2.96		74.00	59.93–88.07	0.59
	Overdominant											
	CC-TT	165 (74.3)	340 (71.6)	1.00	-	0.45	1.00	-	**0.045**	72.00	70.19–73.81	-
	CT	57 (25.7)	135 (28.4)	0.87	0.61–1.25		**0.62**	**0.38–1.00**		73.00	71.47–74.53	0.16
	Log-additive	-		0.89	0.65–1.21	0.45	0.69	0.46–1.03	0.065	-		
rs9821091	Codominant											
	GG	87 (39.2)	179 (37.7)	1.00	-	0.32	1.00	-	**0.045**	72.00	69.77–74.23	-
	GA	97 (43.7)	232 (48.8)	0.86	0.61–1.22		**0.81**	**0.52–1.28**		73.00	71.43–74.57	0.11
	AA	38 (17.1)	64 (13.5)	1.22	0.76–1.97		**1.75**	**0.95–3.20**		71.00	68.17–73.83	0.16
	Dominant											
	GG	87 (39.2)	179 (37.7)	1.00	-	0.70	1.00	-	0.96	72.00	69.77–74.23	-
	GA-AA	135 (60.8)	296 (62.3)	0.94	0.68–1.30		0.99	0.65–1.50		72.00	70.56–73.44	0.42
	Recessive											
	GG-GA	184 (82.9)	411 (86.5)	1.00	-	0.21	1.00	-	**0.02**	72.00	70.76–73.24	-
	AA	38 (17.1)	64 (16.5)	1.33	0.86–1.12		**1.95**	**1.12–3.40**		**71.00**	**68.17–73.83**	**0.017**
	Overdominant											
	GG-AA	125 (56.3)	243 (51.2)	1.00	-	0.20	1.00	-	0.085	71.00	69.06–72.94	-
	GA	97 (43.7)	232 (48.8)	0.81	0.59–1.12		0.70	0.46–1.05		**73.00**	**71.43–74.57**	**0.018**
	Log-additive	-		1.05	0.83–1.32	0.70	1.19	0.89–1.61	0.24	-		

OR: odds ratio; aOR: odds ratio adjusted for age and gender; CI: Confidence Interval. Values in bold are statistically significant (*p* < 0.05).

**Table 3:** Haplotype frequencies between patients and controls and risk estimates for their involvement in gastric cancer onset.

Gene/Haplotype	% Cases	% Controls	aOR	95% CI	*p* Value
***ABCC4*^£^**					
T-T-A	49.09	42.16	1.00	Reference	-
C-C-A	13.84	16.87	0.67	0.42–1.09	0.11
C-T-A	13.12	12.46	0.94	0.52–1.69	0.83
C-C-G	7.07	9.68	**0.47**	**0.23–0.93**	**0.032**
T-T-G	7.42	7.81	0.52	0.26–1.06	0.074
T-C-A	6.61	8.09	0.70	0.35–1.43	0.33
C-T-G	1.33	2.49	0.56	0.11–2.74	0.47
***SLCO2A1*^¥^**					
C-C-G	45.37	45.31	1.00	Reference	-
C-C-A	26.04	27.74	0.89	0.60–1.32	0.57
C-T-G	9.23	11.12	0.58	0.32–1.07	0.084
T-C-A	6.61	4.98	**2.78**	**1.41–5.48**	**0.0034**
T-C-G	6.44	0.48	0.91	0.44–1.87	0.80

aOR: odds ratio adjusted for age; CI: confidence interval. **^£^** SNPs order: rs1678374-rs11678405-rs1751031. **^¥^** SNPs order: rs10935090-rs11915399-rs9821091. Values in bold are statistically significant (*p* < 0.05).

**Table 4:** MDR analysis for gastric cancer risk prediction, considering the SNPs associated with gastric cancer risk.

	CV Accuracy	CV Consistency	aOR	95% CI	*p* Value
**Best model**					
rs689466	0.621	10/10	2.743	1.967–3.826	<0.0001
age, rs1678374	0.687	5/10	4.953	3.434–7.143	<0.0001
age, rs689466, rs1678374	0.807	8/10	17.581	11.672–26.482	<0.0001

MDR: multifactor dimensionality reduction, CV: cross-validation, aOR: odds ratio adjusted for age; CI: confidence interval.

## Data Availability

Data is contained within the article or supplementary material.

## Supplementary Materials

Supplementary Materials can be found at https://www.mdpi.com/1422-0067/22/2/648/s1.

## Abbreviations

15-PGDH	15-hydroxyprostaglandin dehydrogenase
*ABCC4*	ATP binding cassette subfamily c member 4
*B2M*	beta-2-microglobulin
*CASP8*	caspase 8
cDNA	complementary deoxyribonucleic acid
CEU	Utah residents with Northern and Western European ancestry
CI	confidence interval
C_T_	cycle threshold
CVC	cross-variation consistency
EM	expectation-maximization algorithm
FDR	false discovery rate
FFPE	formalin-fixed paraffin-embedded
GC	gastric cancer
*GSTP1*	glutathione s-transferase pi 1
*GUSB*	glucuronidase beta
*HPGD*	15-hydroxyprostaglandin dehydrogenase
*HPRT1*	hypoxanthine phosphoribosyltransferase 1
HWE	Hardy-Weinberg equilibrium
*IPO8*	importin 8
MDR	multifactor dimensionality reduction
mRNA	messenger ribonucleic acid
*MRP4*	multidrug resistance protein 4
*MTX1*	metaxin 1
*MUC1*	mucin 1, cell surface-associated
OD	optical density
PCR	polymerase chain reaction
PGE_2_	prostaglandin E_2_
PGT	prostaglandin transporter
*PKLR*	pyruvate kinase L/R
*PLCE1*	phospholipase C epsilon 1
*PPIA*	peptidylprolyl isomerase A
*PRKAA1*	protein kinase AMP-activated catalytic subunit alpha
*PSCA*	prostate stem cell antigen
*PTGS2*	prostaglandin-endoperoxide synthase 2
*RPL29*	ribosomal protein L29
RT	reverse transcription
*SLCO2A1*	solute carrier organic anion transporter family member 2A1
SPSS	statistical package for the social sciences
*TGFBR2*	transforming growth factor-beta receptor 2
*TNF*	tumor necrosis factor-alpha
