# Supplementary material for: Genetic Variations in Prostaglandin E2 Pathway Identified as Susceptibility Biomarkers for Gastric Cancer in an Intermediate Risk European Country
Source: Int J Mol Sci. 2021 Jan 11;22(2):648. doi: 10.3390/ijms22020648 (PMC7827533; doi:10.3390/ijms22020648)
Supplement: Supplementary file 1 [file ijms-22-00648-s001.pdf]

## SUPPLEMENTARY DATA

**Table S1.** Genetic polymorphisms in *PTGS2*, *ABCC4*, *HPGD*, and *SLCO2A1* genes and quality control results.

| Gene | TagSNP | Other SNPs on the block | Genotype call rate (%) | Genotype concordance rate | HWE | Passed quality check? |
|---|---|---|---|---|---|---|
| ***PTGS2*** | rs5275 | Candidate SNP | 100 | 1.00 | 1.00 | Yes |
| | rs20417 | Candidate SNP | 100 | 1.00 | 1.00 | Yes |
| | rs689466 | Candidate SNP | 100 | 1.00 | 0.52 | Yes |
| ***ABCC4*** | rs12867485 | rs9561811<br>rs17189481 | 0 | - | - | No |
| | rs1611822 | rs1751015 | 100 | 1.00 | 0.85 | Yes |
| | rs1628382 | rs4148527<br>rs8001657<br>rs12584534 | 27 | - | - | No |
| | rs1678354 | rs1751059<br>rs1729759 | 100 | 1.00 | <0.0001 | No |
| | rs1678374 | rs1751025 | 100 | 1.00 | 0.78 | Yes |
| | rs1678386 | rs9516530 | 100 | 1.00 | 0.51 | Yes |
| | rs1678396 | singleton | 100 | 1.00 | 0.26 | Yes |
| | rs1678405 | rs6492768 | 99 | 1.00 | 0.42 | Yes |
| | rs17268122 | rs1479389<br>rs17189376<br>rs17189446<br>rs17268163 | 99 | 1.00 | 0.014 | No |
| | rs1751027 | rs1564351<br>rs4148487<br>rs17189390<br>rs17268129<br>rs17268170 | 100 | 1.00 | 0.23 | Yes |
| | rs1751031 | rs931111<br>rs1189442<br>rs1189443<br>rs1189444<br>rs1189451<br>rs1189452<br>rs1612388<br>rs1630901<br>rs1729747<br>rs1751033<br>rs1751034<br>rs2619312 | 100 | 1.00 | 0.20 | Yes |
| | rs1751051 | rs1751050 | 100 | 1.00 | 1.00 | Yes |
| | rs2127295 | rs1564354<br>rs1564355<br>rs1617785<br>rs1630807<br>rs1638342<br>rs1678363<br>rs1678394<br>rs1729748<br>rs2698243<br>rs2766481<br>rs3825415<br>rs6650282 | 100 | 1.00 | 0.40 | Yes |
| | rs2274403 | rs3864997<br>rs4148481 | 100 | 1.00 | 0.52 | Yes |
| | rs2892713 | rs12865305 | 100 | 1.00 | 0.14 | Yes |
| | rs2892715 | rs9561814 | 100 | 1.00 | 0.26 | Yes |

SNP: Single Nucleotide Polymorphism; HWE: Hardy-Weinberg equilibrium.

**Table S1 (cont.).** Genetic polymorphisms in *PTGS2*, *ABCC4*, *HPGD*, and *SLCO2A1* genes and quality control results.

| Gene | TagSNP | Other SNPs on the block | Genotype call rate (%) | Genotype concordance rate | HWE | Passed quality check? |
|---|---|---|---|---|---|---|
| *ABCC4* | rs3742106 | rs4148544<br>rs4148549<br>rs4148551<br>rs7330196<br>rs9302039<br>rs9524769 | 100 | 1.00 | 0.92 | Yes |
| | rs3782958 | rs4148515<br>rs7985457<br>rs10508023 | 100 | 1.00 | 0.74 | Yes |
| | rs4148421 | singleton | 100 | 1.00 | 1.00 | Yes |
| | rs4148422 | rs17300935 | 100 | 1.00 | 0.0022 | No |
| | rs4148437 | rs2892716<br>rs4148436<br>rs4148446<br>rs9556466<br>rs10508018 | 100 | 1.00 | 0.38 | Yes |
| | rs4148476 | rs4773843<br>rs9524822 | 100 | 1.00 | 0.13 | Yes |
| | rs4612933 | rs899494<br>rs899495<br>rs899496<br>rs1678403<br>rs1824911<br>rs1824913<br>rs1926657<br>rs3782965<br>rs4148465<br>rs4148469<br>rs4303338<br>rs4334136<br>rs4505186<br>rs4773854<br>rs4773855<br>rs7325019<br>rs7333234<br>rs7335147<br>rs7983336<br>rs7987653<br>rs7988494<br>rs9524831<br>rs9524833<br>rs9524845<br>rs9524856<br>rs12870204 | 100 | 1.00 | 1.00 | Yes |
| | rs4771912 | rs7981095 | 100 | 1.00 | 0.70 | Yes |
| | rs6492763 | rs10508024 | 100 | 1.00 | 0.34 | Yes |
| | rs7993878 | rs9302040<br>rs9302042<br>rs9302043<br>rs9556455<br>rs9561768<br>rs9561769<br>rs9590168<br>rs10219913 | 100 | 1.00 | 0.25 | Yes |

SNP: Single Nucleotide Polymorphism; HWE: Hardy-Weinberg equilibrium.

**Table S1 (cont.).** Genetic polymorphisms in *PTGS2*, *ABCC4*, *HPGD*, and *SLCO2A1* genes and quality control results.

| Gene | TagSNP | Other SNPs on the block | Genotype call rate (%) | Genotype concordance rate | HWE | Passed quality check? |
|---|---|---|---|---|---|---|
| ***ABCC4*** | rs8002180 | rs4148424<br>rs4771910<br>rs7317112<br>rs7322318<br>rs8001475<br>rs9584288<br>rs9590228 | 100 | 1.00 | 0.43 | Yes |
| | rs869951 | rs871052 | 100 | 1.00 | 0.70 | Yes |
| | rs9524821 | rs9516532 | 100 | 1.00 | 0.42 | Yes |
| | rs9590220 | rs9590216<br>rs12584649<br>rs17235152 | 100 | 1.00 | 0.016 | No |
| | rs9590222 | rs12100301 | 64 | - | - | No |
| ***HPGD*** | rs2612656 | Candidate SNP | 100 | 1.00 | 0.65 | Yes |
| | rs2555639 | Candidate SNP | 100 | 1.00 | 1.00 | Yes |
| | rs12500316 | rs1863641<br>rs11722919 | 100 | 1.00 | 0.28 | Yes |
| | rs1346271 | singleton | 100 | 1.00 | 0.065 | Yes |
| | rs1426945 | rs3756273 | 100 | 1.00 | 0.92 | Yes |
| | rs1863642 | rs2612659 | 100 | 1.00 | 0.23 | Yes |
| | rs2303520 | rs9312555<br>rs13106936<br>rs13127058<br>rs17060521 | 100 | 1.00 | 0.49 | Yes |
| | rs2555632 | rs3101255 | 100 | 1.00 | 0.43 | Yes |
| | rs2612656 | rs1816204<br>rs3846298<br>rs3857075<br>rs7685956<br>rs11133042 | 0 | - | - | No |
| | rs8752 | rs1426947<br>rs2612658<br>rs11133041<br>rs11724251 | 100 | 1.00 | 0.84 | Yes |
| ***SLCO2A1*** | rs10935090 | singleton | 100 | 1.00 | 0.48 | Yes |
| | rs1131598 | singleton | 100 | 1.00 | 0.26 | Yes |
| | rs11915399 | singleton | 100 | 1.00 | 1.00 | Yes |
| | rs4241362 | rs4241361<br>rs4634113<br>rs6804798<br>rs9828294<br>rs9855403<br>rs9874493<br>rs9882333<br>rs11720811 | 100 | 1.00 | 0.42 | Yes |
| | rs4241365 | rs7653639 | 100 | 1.00 | 0.61 | Yes |
| | rs4331673 | rs4241361<br>rs6804798<br>rs9828294<br>rs9840711<br>rs9855403<br>rs9857498<br>rs9874493<br>rs11720811<br>rs11720843 | 100 | 1.00 | 0.87 | Yes |
| | rs4854784 | singleton | 100 | 1.00 | 0.61 | Yes |

SNP: Single Nucleotide Polymorphism; HWE: Hardy-Weinberg equilibrium.

**Table S1 (cont.).** Genetic polymorphisms in *PTGS2*, *ABCC4*, *HPGD*, and *SLCO2A1* genes and quality control results.

| Gene | TagSNP | Other SNPs on the block | Genotype call rate (%) | Genotype concordance rate | HWE | Passed quality check? |
|---|---|---|---|---|---|---|
| ***SLCO2A1*** | rs6439448 | rs2370512<br>rs3923835<br>rs34550074 | 100 | 1.00 | 0.88 | Yes |
| | rs7340717 | rs7340718 | 100 | 1.00 | 0.32 | Yes |
| | rs7616492 | rs10935089 | 100 | 1.00 | 0.69 | Yes |
| | rs7625035 | rs9822027 | 100 | 1.00 | 0.30 | Yes |
| | rs7646392 | rs4327389<br>rs7646298<br>rs7646473 | 100 | 1.00 | 0.29 | Yes |
| | rs9820625 | rs9836830<br>rs9917636<br>rs11709172<br>rs13083175 | 100 | 1.00 | 0.46 | Yes |
| | rs9821091 | rs6439450<br>rs7617777<br>rs9834727<br>rs9841380 | 100 | 1.00 | 0.44 | Yes |
| | rs9834412 | rs4854785<br>rs13067921 | 100 | 1.00 | 0.90 | Yes |

SNP: Single Nucleotide Polymorphism; HWE: Hardy-Weinberg equilibrium.

**Table S2.** Genotype risk estimates for the involvement of *PTGS2*, *ABCC4*, *HPGD*, and *SLCO2A1* genetic variants in gastric cancer onset and estimated age at diagnosis.

| SNP | Model | Genotype | Univariate analysis | | | Multivariate analysis* | | | Age at diagnosis | | |
|---|---|---|---|---|---|---|---|---|---|---|---|
| | | | OR | 95% CI | p value | aOR | 95% CI | p value | Median (years) | 95% CI | p value |
| ***PTGS2*** | | | | | | | | | | | |
| rs5275 | Codominant | TT | 1.00 | - | 0.50 | 1.00 | - | 0.57 | 73.00 | 71.36-74.64 | - |
| | | TC | 1.20 | 0.85-1.71 | | 1.27 | 0.81-1.99 | | 73.00 | 70.58-75.42 | 0.18 |
| | | CC | 1.28 | 0.70-2.34 | | 1.20 | 0.55-2.63 | | 76.00 | 70.72-81.28 | 0.47 |
| | Dominant | TT | 1.00 | - | 0.25 | 1.00 | - | 0.29 | 73.00 | 71.36-74.64 | - |
| | | TC-CC | 1.22 | 0.87-1.70 | | 1.26 | 0.82-1.93 | | 73.00 | 70.58-75.42 | 0.43 |
| | Recessive | TT-TC | 1.00 | - | 0.59 | 1.00 | - | 0.85 | 73.00 | 71.62-74.38 | - |
| | | CC | 1.17 | 0.66-2.09 | | 1.08 | 0.51-2.28 | | 76.00 | 70.72-81.28 | 0.25 |
| | Overdominant | TT-CC | 1.00 | - | 0.39 | 1.00 | - | 0.34 | 73.00 | 71.32-74.68 | - |
| | | TC | 1.16 | 0.83-1.62 | | 1.24 | 0.80-1.90 | | 73.00 | 71.58-75.42 | 0.12 |
| | Log-additive | - | 1.16 | 0.90-1.50 | 0.26 | 1.16 | 0.84-1.62 | 0.37 | - | | |
| rs20417 | Codominant | GG | 1.00 | - | 0.76 | 1.00 | - | 0.65 | 73.00 | 71.60-74.40 | - |
| | | GC | 1.15 | 0.79-1.65 | | 1.17 | 0.73-1.87 | | 72.00 | 69.73-74.27 | 0.26 |
| | | CC | 1.15 | 0.43-3.10 | | 1.61 | 0.46-5.57 | | 77.00 | 57.75-96.25 | 0.53 |
| | Dominant | GG | 1.00 | - | 0.45 | 1.00 | - | 0.42 | 73.00 | 71.60-74.40 | - |
| | | GC-CC | 1.15 | 0.80-1.63 | | 1.20 | 0.76-1.89 | | 72.00 | 68.91-75.09 | 0.43 |
| | Recessive | GG-GC | 1.00 | - | 0.84 | 1.00 | - | 0.51 | 73.00 | 71.63-74.37 | - |
| | | CC | 1.11 | 1.41-2.96 | | 1.53 | 0.44-5.26 | | 77.00 | 57.75-96.25 | 0.44 |
| | Overdominant | GG-CC | 1.00 | - | 0.49 | 1.00 | - | 0.56 | 73.00 | 71.58-74.42 | - |
| | | GC | 1.14 | 0.79-1.64 | | 1.15 | 0.72-1.83 | | 72.00 | 69.73-74.27 | 0.26 |
| | Log-additive | - | 1.12 | 0.82-1.53 | 0.47 | 1.20 | 0.81-1.79 | 0.36 | - | | |
| rs689466 | Codominant | AA | 1.00 | - | 0.054 | 1.00 | - | **0.021** | 73.00 | 71.25-74.75 | - |
| | | AG | 1.29 | 0.89-1.86 | | 1.50 | 0.93-2.42 | | 73.00 | 68.89-77.11 | 0.21 |
| | | GG | **2.33** | **1.10-4.92** | | **3.40** | **1.29-8.97** | | **70.00** | **62.49-77.52** | **0.008** |
| | Dominant | AA | 1.00 | - | 0.056 | 1.00 | - | **0.022** | 73.00 | 71.25-74.75 | - |
| | | AG-GG | 1.40 | 0.99-1.98 | | **1.69** | **1.08-2.65** | | 73.00 | 69.94-76.06 | 0.058 |
| | Recessive | AA-AG | 1.00 | - | **0.046** | 1.00 | - | **0.027** | 73.00 | 71.48-74.52 | - |
| | | GG | **2.15** | **1.03-4.49** | | **2.98** | **1.14-7.74** | | **70.00** | **62.49-77.52** | **0.011** |
| | Overdominant | AA-GG | 1.00 | - | 0.30 | 1.00 | - | 0.19 | 73.00 | 71.46-74.54 | - |
| | | AG | 1.21 | 0.85-1.74 | | 1.37 | 0.86-2.19 | | 73.00 | 68.89-77.11 | 0.38 |
| | Log-additive | - | **1.40** | **1.06-1.86** | **0.021** | **1.66** | **1.15-2.40** | **0.007** | - | | |

OR: odds ratio; aOR: odds ratio adjusted for age and gender; CI: confidence interval.
Values in **bold** are statistically significant ($p < 0.05$).

**Table S2 (cont.).** Genotype risk estimates for the involvement of *PTGS2*, *ABCC4*, *HPGD*, and *SLCO2A1* genetic variants in gastric cancer onset and estimated age at diagnosis.

| SNP | Model | Genotype | Univariate analysis | | | Multivariate analysis* | | | Age at diagnosis | | |
|---|---|---|---|---|---|---|---|---|---|---|---|
| | | | OR | 95% CI | p value | aOR | 95% CI | p value | Median (years) | 95% CI | p value |
| ***ABCC4*** | | | | | | | | | | | |
| rs1611822 | Codominant | CC | 1.00 | - | 0.20 | 1.00 | - | 0.53 | 72.00 | 70.49-73.51 | - |
| | | CT | 1.24 | 0.87-1.78 | | 1.22 | 0.77-1.93 | | 72.00 | 70.01-73.99 | 0.95 |
| | | TT | 1.52 | 0.95-2.43 | | 1.37 | 0.75-2.47 | | 72.00 | 66.99-77.01 | 0.42 |
| | Dominant | CC | 1.00 | - | 0.12 | 1.00 | - | 0.30 | 72.00 | 70.49-73.51 | - |
| | | CT-TT | 1.31 | 0.93-1.83 | | 1.26 | 0.82-1.93 | | 72.00 | 70.24-73.76 | 0.75 |
| | Recessive | CC-CT | 1.00 | - | 0.18 | 1.00 | - | 0.46 | 72.00 | 70.80-73.20 | - |
| | | TT | 1.34 | 0.88-2.04 | | 1.22 | 0.72-2.08 | | 72.00 | 66.99-77.01 | 0.45 |
| | Overdominant | CC-TT | 1.00 | - | 0.62 | 1.00 | - | 0.66 | 72.00 | 70.55-73.45 | - |
| | | CT | 1.08 | 0.79-1.49 | | 1.10 | 0.73-1.65 | | 72.00 | 70.01-73.99 | 0.78 |
| | Log-additive | - | 1.23 | 0.98-1.55 | 0.072 | 1.18 | 0.88-1.57 | 0.27 | - | | |
| rs1678374 | Codominant | TT | 1.00 | - | 0.076 | 1.00 | - | 0.063 | 72.00 | 69.48-74.52 | - |
| | | TC | 0.82 | 0.58-1.15 | | 1.04 | 0.67-1.63 | | 72.00 | 69.74-74.26 | 0.47 |
| | | CC | **0.56** | **0.33-0.94** | | **0.50** | **0.26-0.97** | | 73.00 | 71.00-75.00 | 0.57 |
| | Dominant | TT | 1.00 | - | 0.09 | 1.00 | - | 0.55 | 72.00 | 69.48-74.52 | - |
| | | TC-CC | 0.75 | 0.54-1.04 | | 0.88 | 0.58-1.34 | | 72.00 | 70.77-73.23 | 0.66 |
| | Recessive | TT-TC | 1.00 | - | 0.05 | 1.00 | - | **0.019** | 72.00 | 70.38-73.63 | - |
| | | CC | 0.63 | 0.39-1.01 | | **0.49** | **0.26-0.91** | | 73.00 | 71.00-75.00 | 0.39 |
| | Overdominant | TT-CC | 1.00 | - | 0.30 | 1.00 | - | 0.28 | 72.00 | 70.46-73.54 | - |
| | | TC | 1.21 | 0.85-1.74 | | 1.25 | 0.83-1.89 | | 72.00 | 69.74-74.26 | 0.31 |
| | Log-additive | - | **0.77** | **0.60-0.97** | **0.027** | 0.78 | 0.58-1.06 | 0.11 | - | | |
| rs1678386 | Codominant | AA | 1.00 | - | 0.82 | 1.00 | - | 0.34 | 72.00 | 70.04-73.96 | - |
| | | AC | 0.90 | 0.64-1.26 | | 0.75 | 0.49-1.16 | | 72.00 | 70.48-73.52 | 0.80 |
| | | CC | 0.98 | 0.56-1.72 | | 1.13 | 0.55-2.33 | | 71.00 | 58.59-83.41 | 0.87 |
| | Dominant | AA | 1.00 | - | 0.57 | 1.00 | - | 0.31 | 72.00 | 70.04-73.96 | - |
| | | AC-CC | 0.91 | 0.66-1.26 | | 0.81 | 0.54-1.22 | | 72.00 | 70.06-73.94 | 0.89 |
| | Recessive | AA-AC | 1.00 | - | 0.93 | 1.00 | - | 0.50 | 72.00 | 70.91-73.09 | - |
| | | CC | 1.03 | 0.59-1.77 | | 1.28 | 0.63-2.58 | | 71.00 | 58.59-83.41 | 0.75 |
| | Overdominant | AA-CC | 1.00 | - | 0.53 | 1.00 | - | 0.15 | 72.00 | 70.03-73.97 | - |
| | | AC | 0.90 | 0.65-1.25 | | 0.74 | 0.48-1.12 | | 72.00 | 70.48-73.52 | 0.74 |
| | Log-additive | - | 0.95 | 0.75-1.22 | 0.70 | 0.92 | 0.67-1.27 | 0.63 | - | | |

OR: odds ratio; aOR: odds ratio adjusted for age and gender; CI: confidence interval.
Values in **bold** are statistically significant ($p < 0.05$).

**Table S2 (cont.).** Genotype risk estimates for the involvement of *PTGS2*, *ABCC4*, *HPGD*, and *SLCO2A1* genetic variants in gastric cancer onset and estimated age at diagnosis.

| SNP | Model | Genotype | Univariate analysis | | | Multivariate analysis* | | | Age at diagnosis | | |
|---|---|---|---|---|---|---|---|---|---|---|---|
| | | | OR | 95% CI | p value | aOR | 95% CI | p value | Median (years) | 95% CI | p value |
| rs1678396 | Codominant | TT | 1.00 | - | 0.74 | 1.00 | - | 0.77 | 70.00 | 68.08-71.92 | - |
| | | TC | 0.91 | 0.63-1.30 | | 0.88 | 0.55-1.40 | | 74.00 | 71.50-76.50 | 0.39 |
| | | CC | 1.06 | 0.67-1.69 | | 0.82 | 0.45-1.47 | | 72.00 | 69.70-74.30 | 0.58 |
| | Dominant | TT | 1.00 | - | 0.75 | 1.00 | - | 0.50 | 70.00 | 68.08-71.92 | - |
| | | TC-CC | 0.94 | 0.67-1.33 | | 0.86 | 0.56-1.33 | | 73.00 | 71.65-74.35 | 0.39 |
| | Recessive | TT-TC | 1.00 | - | 0.57 | 1.00 | - | 0.63 | 72.00 | 70.67-73.33 | - |
| | | CC | 1.13 | 0.75-1.70 | | 0.88 | 0.52-1.48 | | 72.00 | 69.70-74.30 | 0.92 |
| | Overdominant | TT-CC | 1.00 | - | 0.46 | 1.00 | - | 0.81 | 71.00 | 69.65-72.35 | - |
| | | TC | 0.89 | 0.64-1.22 | | 0.95 | 0.63-1.43 | | 74.00 | 71.50-76.50 | 0.39 |
| | Log-additive | - | 1.01 | 0.80-1.28 | 0.92 | 0.90 | 0.67-1.20 | 0.48 | - | | |
| rs1678405 | Codominant | TT | 1.00 | - | 0.052 | 1.00 | - | **0.09** | 72.00 | 70.40-73.60 | - |
| | | TC | 0.73 | 0.52-1.02 | | 0.81 | 0.52-1.25 | | 72.00 | 69.60-74.40 | 0.58 |
| | | CC | **0.54** | **0.29-0.99** | | **0.44** | **0.20-0.95** | | 74.00 | 67.60-80.40 | 0.68 |
| | Dominant | TT | 1.00 | - | **0.027** | 1.00 | - | 0.13 | 72.00 | 70.40-73.60 | - |
| | | TC-CC | **0.69** | **0.50-0.96** | | 0.73 | 0.48-1.10 | | 72.00 | 70.18-73.82 | 0.70 |
| | Recessive | TT-TC | 1.00 | - | 0.11 | 1.00 | - | **0.049** | 72.00 | 70.58-73.42 | - |
| | | CC | 0.63 | 0.35-1.13 | | **0.49** | **0.23-1.03** | | 74.00 | 67.60-80.40 | 0.58 |
| | Overdominant | TT-CC | 1.00 | - | 0.21 | 1.00 | - | 0.76 | 72.00 | 70.46-73.54 | - |
| | | TC | 0.81 | 0.59-1.12 | | 0.94 | 0.62-1.42 | | 72.00 | 69.60-74.40 | 0.48 |
| | Log-additive | - | **0.73** | **0.57-0.94** | **0.015** | **0.72** | **0.52-0.99** | **0.041** | | - | |
| rs1751027 | Codominant | AA | 1.00 | - | 0.096 | 1.00 | - | 0.66 | 72.00 | 70.19-73.81 | - |
| | | AG | 1.30 | 0.86-1.96 | | 1.27 | 0.73-2.20 | | 72.00 | 71.05-72.95 | 0.51 |
| | | GG | 6.82 | 0.70-66.05 | | 1.64 | 0.12-22.90 | | 73.00 | 66.60-79.40 | 0.96 |
| | Dominant | AA | 1.00 | - | 0.13 | 1.00 | - | 0.37 | 72.00 | 70.19-73.81 | - |
| | | AG-GG | 1.37 | 0.92-2.05 | | 1.28 | 0.75-2.20 | | 72.00 | 71.00-73.00 | 0.52 |
| | Recessive | AA-AG | 1.00 | - | 0.076 | 1.00 | - | 0.73 | 72.00 | 70.58-73.42 | - |
| | | GG | 6.51 | 0.67-62.91 | | 1.57 | 0.11-22.03 | | 73.00 | 66.60-79.40 | 1.00 |
| | Overdominant | AA-GG | 1.00 | - | 0.24 | 1.00 | - | 0.40 | 72.00 | 70.26-73.74 | - |
| | | AG | 1.28 | 0.85-1.93 | | 1.27 | 0.73-2.19 | | 72.00 | 71.05-72.95 | 0.51 |
| | Log-additive | - | 1.42 | 0.97-2.08 | 0.074 | 1.27 | 0.76-2.12 | 0.36 | | - | |

OR: odds ratio; aOR: odds ratio adjusted for age and gender; CI: confidence interval.
Values in **bold** are statistically significant ($p < 0.05$).

**Table S2 (cont.).** Genotype risk estimates for the involvement of *PTGS2*, *ABCC4*, *HPGD*, and *SLCO2A1* genetic variants in gastric cancer onset and estimated age at diagnosis.

| SNP | Model | Genotype | Univariate analysis | | | Multivariate analysis* | | | Age at diagnosis | | |
|---|---|---|---|---|---|---|---|---|---|---|---|
| | | | OR | 95% CI | p value | aOR | 95% CI | p value | Median (years) | 95% CI | p value |
| rs1751031 | Codominant | AA | 1.00 | - | 0.10 | 1.00 | - | 0.073 | 72.00 | 70.25-73.75 | - |
| | | AG | **0.69** | **0.48** | | **0.61** | **0.39-0.95** | | 72.00 | 69.72-74.28 | 0.66 |
| | | GG | 1.15 | 0.49-2.70 | | 0.57 | 0.17-1.92 | | 78.00 | 69.81-86.19 | 0.29 |
| | Dominant | AA | 1.00 | - | 0.068 | 1.00 | - | **0.022** | 72.00 | 70.25-73.75 | **-** |
| | | AG-GG | 0.73 | 0.52-1.03 | | **0.60** | **0.39-0.94** | | 72.00 | 70.08-73.93 | 0.46 |
| | Recessive | AA-AG | 1.00 | - | 0.55 | 1.00 | - | 0.52 | 72.00 | 70.61-73.39 | - |
| | | GG | 1.30 | 0.56-3.01 | | 0.68 | 0.21-2.24 | | 78.00 | 69.81-86.19 | 0.31 |
| | Overdominant | AA-GG | 1.00 | - | **0.034** | 1.00 | - | **0.036** | 72.00 | 70.28-73.72 | **-** |
| | | AG | **0.69** | **0.48-0.98** | | **0.62** | **0.40-0.98** | | 72.00 | 69.72-74.28 | 0.79 |
| | Log-additive | - | 0.81 | 0.61-1.09 | 0.17 | **0.65** | **0.44-0.96** | **0.028** | **-** | | |
| rs1751051 | Codominant | TT | 1.00 | - | 0.058 | 1.00 | - | 0.22 | 71.00 | 69.28-72.72 | - |
| | | TA | **1.50** | **1.07-2.10** | | 1.39 | 0.90-2.14 | | 73.00 | 71.46-74.54 | 0.96 |
| | | AA | 1.36 | 0.77-2.41 | | 1.61 | 0.80-3.21 | | 71.00 | 65.65-76.35 | 0.54 |
| | Dominant | TT | 1.00 | - | **0.018** | 1.00 | - | 0.09 | 73.00 | 71.45-74.55 | - |
| | | TA-AA | **1.48** | **1.07-2.04** | | 1.43 | 0.94-2.16 | | 71.00 | 69.28-72.72 | 0.86 |
| | Recessive | TT-TA | 1.00 | - | 0.71 | 1.00 | - | 0.35 | 72.00 | 70.85-73.15 | - |
| | | AA | 1.11 | 0.65-1.90 | | 1.37 | 0.71-2.64 | | 71.00 | 65.65-76.35 | 0.49 |
| | Overdominant | TT-AA | 1.00 | - | **0.032** | 1.00 | - | 0.26 | 71.00 | 69.38-72.62 | - |
| | | TA | **1.42** | **1.03-1.95** | | 1.27 | 0.84-1.92 | | 73.00 | 71.46-74.54 | 0.83 |
| | Log-additive | - | **1.28** | **1.00-1.64** | **0.048** | 1.31 | 0.96-1.78 | 0.088 | - | | |
| rs2127295 | Codominant | GG | 1.00 | - | 0.39 | 1.00 | - | 0.30 | 73.00 | 71.01-74.99 | - |
| | | GA | 0.94 | 0.64-1.36 | | 1.10 | 0.67-1.81 | | 71.00 | 69.16-72.84 | 0.61 |
| | | AA | 1.24 | 0.79-1.95 | | 1.55 | 0.86-2.79 | | 72.00 | 69.64-74.36 | 0.50 |
| | Dominant | GG | 1.00 | - | 0.91 | 1.00 | - | 0.39 | 73.00 | 71.01-74.99 | - |
| | | GA-AA | 1.02 | 0.72-1.45 | | 1.22 | 0.77-1.95 | | 72.00 | 70.40-73.60 | 0.52 |
| | Recessive | GG-GA | 1.00 | - | 0.18 | 1.00 | - | 0.14 | 72.00 | 70.51-73.49 | - |
| | | AA | 1.30 | 0.89-1.90 | | 1.46 | 0.89-2.37 | | 72.00 | 69.64-74.36 | 0.49 |
| | Overdominant | GG-AA | 1.00 | - | 0.32 | 1.00 | - | 0.64 | 72.00 | 70.44-73.56 | - |
| | | GA | 0.85 | 0.62-1.17 | | 0.91 | 0.60-1.37 | | 71.00 | 69.16-72.84 | 0.98 |
| | Log-additive | - | 1.10 | 0.88-1.39 | 0.39 | 1.24 | 0.92-1.67 | 0.15 | - | | |

OR: odds ratio; aOR: odds ratio adjusted for age and gender; CI: confidence interval.
Values in **bold** are statistically significant ($p < 0.05$).

**Table S2 (cont.).** Genotype risk estimates for the involvement of *PTGS2*, *ABCC4*, *HPGD*, and *SLCO2A1* genetic variants in gastric cancer onset and estimated age at diagnosis.

| SNP | Model | Genotype | Univariate analysis | | | Multivariate analysis* | | | Age at diagnosis | | |
|---|---|---|---|---|---|---|---|---|---|---|---|
| | | | OR | 95% CI | p value | aOR | 95% CI | p value | Median (years) | 95% CI | p value |
| rs2274403 | Codominant | AA | 1.00 | - | **0.016** | 1.00 | - | 0.41 | 72.00 | 70.39-73.61 | - |
| | | AG | 0.78 | 0.54-1.13 | | 0.92 | 0.58-1.49 | | 73.00 | 71.14-74.86 | 0.42 |
| | | GG | **0.51** | **0.32-0.81** | | 0.69 | 0.39-1.22 | | 73.00 | 70.91-75.09 | 0.93 |
| | Dominant | AA | 1.00 | - | **0.035** | 1.00 | - | 0.44 | 72.00 | 70.39-73.61 | - |
| | | AG-GG | **0.69** | **0.48-0.97** | | 0.84 | 0.54-1.31 | | 73.00 | 71.56-74.44 | 0.52 |
| | Recessive | AA-AG | 1.00 | - | **0.01** | 1.00 | - | 0.20 | 72.00 | 70.40-73.60 | - |
| | | GG | **0.60** | **0.40-0.89** | | 0.72 | 0.44-1.19 | | 73.00 | 70.91-75.09 | 0.56 |
| | Overdominant | AA-GG | 1.00 | - | 0.80 | 1.00 | - | 0.70 | 72.00 | 70.75-73.25 | - |
| | | AG | 1.04 | 0.76-1.43 | | 1.08 | 0.72-1.63 | | 73.00 | 71.14-74.86 | 0.31 |
| | Log-additive | - | **0.72** | **0.57-0.91** | **0.0046** | 0.84 | 0.63-1.11 | 0.22 | - | | |
| rs2892713 | Codominant | CC | 1.00 | - | 0.71 | 1.00 | - | 0.53 | 72.00 | 70.22-73.78 | - |
| | | CT | 1.07 | 0.74-1.53 | | 0.91 | 0.57-1.45 | | 72.00 | 71.16-72.85 | 0.60 |
| | | TT | 0.72 | 0.28-1.85 | | 0.51 | 0.15-1.79 | | 75.00 | 66.16-83.84 | 0.44 |
| | Dominant | CC | 1.00 | - | 0.89 | 1.00 | - | 0.50 | 72.00 | 70.22-73.78 | - |
| | | CT-TT | 1.02 | 0.72-1.45 | | 0.86 | 0.55-1.35 | | 72.00 | 71.11-72.89 | 0.48 |
| | Recessive | CC-CT | 1.00 | - | 0.45 | 1.00 | - | 0.29 | 72.00 | 70.64-73.36 | - |
| | | TT | 0.71 | 0.28-1.80 | | 0.53 | 0.15-1.82 | | 75.00 | 66.16-83.84 | 0.48 |
| | Overdominant | CC-TT | 1.00 | - | 0.66 | 1.00 | - | 0.79 | 72.00 | 70.17-73.83 | - |
| | | CT | 1.08 | 0.76-1.55 | | 0.94 | 0.59-1.49 | | 72.00 | 71.16-72.85 | 0.66 |
| | Log-additive | - | 0.98 | 0.73-1.32 | 0.89 | 0.84 | 0.57-1.23 | 0.36 | | - | |
| rs2892715 | Codominant | GG | 1.00 | - | 0.52 | 1.00 | - | 0.73 | 72.00 | 70.56-73.44 | - |
| | | GA | 1.07 | 0.75-1.53 | | 1.18 | 0.75-1.85 | | 73.00 | 71.20-74.80 | 0.56 |
| | | AA | 0.82 | 0.51-1.33 | | 0.99 | 0.54-1.81 | | 72.00 | 69.81-74.19 | 0.76 |
| | Dominant | GG | 1.00 | - | 1.00 | 1.00 | - | 0.60 | 72.00 | 70.56-73.44 | - |
| | | GA-AA | 1.00 | 0.72-1.40 | | 1.12 | 0.73-1.72 | | 73.00 | 71.58-74.42 | 0.67 |
| | Recessive | GG-GA | 1.00 | - | 0.28 | 1.00 | - | 0.71 | 72.00 | 70.42-73.58 | - |
| | | AA | 0.79 | 0.51-1.22 | | 0.90 | 0.52-1.56 | | 72.00 | 69.81-74.19 | 0.56 |
| | Overdominant | GG-AA | 1.00 | - | 0.42 | 1.00 | - | 0.43 | 72.00 | 70.79-73.21 | - |
| | | GA | 1.14 | 0.83-1.57 | | 1.18 | 0.78-1.78 | | 73.00 | 71.20-74.80 | 0.42 |
| | Log-additive | - | 0.94 | 0.75-1.18 | 0.57 | 1.02 | 0.77-1.37 | 0.87 | | - | |

OR: odds ratio; aOR: odds ratio adjusted for age and gender; CI: confidence interval.
Values in **bold** are statistically significant ($p < 0.05$).

**Table S2 (cont.).** Genotype risk estimates for the involvement of *PTGS2*, *ABCC4*, *HPGD*, and *SLCO2A1* genetic variants in gastric cancer onset and estimated age at diagnosis.

| SNP | Model | Genotype | Univariate analysis | | | Multivariate analysis* | | | Age at diagnosis | | |
|---|---|---|---|---|---|---|---|---|---|---|---|
| | | | OR | 95% CI | p value | aOR | 95% CI | p value | Median (years) | 95% CI | p value |
| rs3742106 | Codominant | AA | 1.00 | - | 0.17 | 1.00 | - | 0.65 | 72.00 | 69.49-74.51 | - |
| | | AC | 1.12 | 0.79-1.58 | | 1.13 | 0.72-1.76 | | 72.00 | 70.22-73.79 | 0.85 |
| | | CC | 0.70 | 0.42-1.18 | | 0.85 | 0.44-1.63 | | 73.00 | 68.95-77.05 | 0.92 |
| | Dominant | AA | 1.00 | - | 0.95 | 1.00 | - | 0.80 | 72.00 | 69.49-74.51 | - |
| | | AC-CC | 1.01 | 0.72-1.41 | | 1.06 | 0.69-1.62 | | 72.00 | 70.78-73.22 | 0.86 |
| | Recessive | AA-AC | 1.00 | - | 0.075 | 1.00 | - | 0.44 | 72.00 | 70.39-73.61 | - |
| | | CC | 0.66 | 0.41-1.06 | | 0.79 | 0.43-1.44 | | 73.00 | 68.95-77.05 | 0.96 |
| | Overdominant | AA-CC | 1.00 | - | 0.19 | 1.00 | - | 0.43 | 72.00 | 69.82-74.18 | - |
| | | AC | 1.24 | 0.90-1.70 | | 1.18 | 0.78-1.78 | | 72.00 | 70.22-73.79 | 0.89 |
| | Log-additive | - | 0.90 | 0.71-1.14 | 0.38 | 0.97 | 0.71-1.31 | 0.83 | - | | |
| rs3782958 | Codominant | GG | 1.00 | - | 0.20 | 1.00 | - | 0.14 | 72.00 | 70.29-73.71 | - |
| | | GC | 0.80 | 0.55-1.16 | | 0.72 | 0.45-1.16 | | 72.00 | 69.75-74.25 | 0.71 |
| | | CC | 0.43 | 0.12-1.50 | | 0.32 | 0.07-1.44 | | 74.00 | - | 0.92 |
| | Dominant | GG | 1.00 | - | 0.14 | 1.00 | - | 0.096 | 72.00 | 70.29-73.71 | - |
| | | GC-CC | 0.76 | 0.53-1.10 | | 0.68 | 0.43-1.08 | | 72.00 | 69.73-74.29 | 0.72 |
| rs3782958 | Recessive | GG-GC | 1.00 | - | 0.18 | 1.00 | - | 0.15 | 72.00 | 70.63-73.38 | - |
| | | CC | 0.45 | 0.13-1.59 | | 0.35 | 0.08-1.57 | | 74.00 | - | 0.96 |
| | Overdominant | GG-CC | 1.00 | - | 0.29 | 1.00 | - | 0.23 | 72.00 | 70.28-73.72 | - |
| | | GC | 0.82 | 0.57-1.19 | | 0.75 | 0.47-1.20 | | 72.00 | 69.75-74.25 | 0.70 |
| | Log-additive | - | 0.76 | 0.55-1.05 | 0.092 | 0.68 | 0.45-1.02 | 0.057 | - | | |
| rs4148421 | Codominant | GG | 1.00 | - | 0.66 | 1.00 | - | 0.27 | 71.00 | 67.11-74.89 | - |
| | | GA | 0.90 | 0.61-1.31 | | 0.69 | 0.43-1.12 | | 72.00 | 70.81-73.19 | 0.81 |
| | | AA | 1.07 | 0.69-1.66 | | 0.92 | 0.52-1.63 | | 72.00 | 69.24-74.76 | 0.62 |
| | Dominant | GG | 1.00 | - | 0.78 | 1.00 | - | 0.23 | 71.00 | 67.11-74.89 | - |
| | | GA-AA | 0.95 | 0.67-1.35 | | 0.76 | 0.48-1.19 | | 72.00 | 70.84-73.16 | 0.71 |
| | Recessive | GG-GA | 1.00 | - | 0.47 | 1.00 | - | 0.55 | 72.00 | 70.54-73.46 | - |
| | | AA | 1.15 | 0.79-1.67 | | 1.16 | 0.71-1.89 | | 72.00 | 69.24-74.76 | 0.63 |
| | Overdominant | GG-AA | 1.00 | - | 0.39 | 1.00 | - | 0.11 | 72.00 | 69.71-74.29 | - |
| | | GA | 0.87 | 0.63-1.20 | | 0.72 | 0.47-1.08 | | 72.00 | 70.81-73.19 | 0.93 |
| | Log-additive | - | 1.03 | 0.82-1.29 | 0.81 | 0.94 | 0.70-1.26 | 0.68 | - | | |

OR: odds ratio; aOR: odds ratio adjusted for age and gender; CI: confidence interval.
Values in **bold** are statistically significant ($p < 0.05$).

**Table S2 (cont.).** Genotype risk estimates for the involvement of *PTGS2*, *ABCC4*, *HPGD*, and *SLCO2A1* genetic variants in gastric cancer onset and estimated age at diagnosis.

| SNP | Model | Genotype | Univariate analysis | | | Multivariate analysis* | | | Age at diagnosis | | |
|---|---|---|---|---|---|---|---|---|---|---|---|
| | | | OR | 95% CI | p value | aOR | 95% CI | p value | Median (years) | 95% CI | p value |
| rs4148437 | Codominant | TT | 1.00 | - | 0.67 | 1.00 | - | 0.87 | 72.00 | 70.47-73.53 | - |
| | | TC | 1.08 | 0.76-1.52 | | 1.09 | 0.70-1.71 | | 73.00 | 71.10-74.91 | 0.38 |
| | | CC | 0.86 | 0.52-1.43 | | 0.95 | 0.51-1.79 | | 72.00 | 66.67-77.33 | 0.65 |
| | Dominant | TT | 1.00 | - | 0.90 | 1.00 | - | 0.79 | 72.00 | 70.47-73.53 | - |
| | | TC-CC | 1.02 | 0.74-1.42 | | 1.06 | 0.70-1.61 | | 73.00 | 71.55-74.45 | 0.52 |
| | Recessive | TT-TC | 1.00 | - | 0.43 | 1.00 | - | 0.74 | 72.00 | 70.47-73.53 | - |
| | | CC | 0.83 | 0.52-1.32 | | 0.91 | 0.51-1.63 | | 72.00 | 66.67-77.33 | 0.45 |
| | Overdominant | TT-CC | 1.00 | - | 0.50 | 1.00 | - | 0.62 | 72.00 | 70.67-73.33 | - |
| | | TC | 1.12 | 0.81-1.54 | | 1.11 | 0.73-1.67 | | 73.00 | 71.10-74.91 | 0.27 |
| | Log-additive | - | 0.96 | 0.76-1.22 | 0.76 | 1.00 | 0.75-1.35 | 0.98 | - | | |
| rs4148476 | Codominant | TT | 1.00 | - | 0.81 | 1.00 | - | 0.44 | 72.00 | 70.67-73.34 | - |
| | | TG | 1.12 | 0.78-1.61 | | 1.27 | 0.79-2.05 | | 74.00 | 70.85-77.15 | 0.57 |
| | | GG | 0.98 | 0.42-2.31 | | 1.66 | 0.58-4.77 | | 71.00 | 55.55-86.46 | 0.61 |
| | Dominant | TT | 1.00 | - | 0.57 | 1.00 | - | 0.23 | 72.00 | 70.67-73.34 | - |
| | | TG-GG | 1.11 | 0.78-1.56 | | 1.32 | 0.84-2.07 | | 73.00 | 69.73-76.27 | 0.69 |
| | Recessive | TT-TG | 1.00 | - | 0.91 | 1.00 | - | 0.42 | 72.00 | 70.88-73.12 | - |
| | | GG | 0.95 | 0.41-2.22 | | 1.56 | 0.55-4.42 | | 71.00 | 55.55-86.46 | 0.57 |
| | Overdominant | TT-GG | 1.00 | - | 0.52 | 1.00 | - | 0.37 | 72.00 | 70.72-73.28 | - |
| | | TG | 1.12 | 0.79-1.61 | | 1.24 | 0.78-1.98 | | 74.00 | 70.85-77.15 | 0.53 |
| | Log-additive | - | 1.07 | 0.80-1.42 | 0.66 | 1.28 | 0.88-1.86 | 0.20 | - | | |
| rs4612933 | Codominant | CC | 1.00 | - | 0.45 | 1.00 | - | 0.31 | 72.00 | 70.39-73.61 | - |
| | | CT | 0.81 | 0.56-1.16 | | 0.73 | 0.46-1.15 | | 72.00 | 69.83-74.17 | 0.44 |
| | | TT | 1.13 | 0.51-2.50 | | 1.31 | 0.48-3.61 | | 67.00 | 58.62-75.38 | 0.68 |
| | Dominant | CC | 1.00 | - | 0.32 | 1.00 | - | 0.27 | 72.00 | 70.39-73.61 | - |
| | | CT-TT | 0.84 | 0.60-1.18 | | 0.78 | 0.51-1.21 | | 72.00 | 70.00-74.00 | 0.56 |
| | Recessive | CC-CT | 1.00 | - | 0.65 | 1.00 | - | 0.48 | 72.00 | 70.60-73.40 | - |
| | | TT | 1.20 | 0.54-2.64 | | 1.44 | 0.53-3.94 | | 67.00 | 58.62-75.38 | 0.57 |
| | Overdominant | CC-TT | 1.00 | - | 0.22 | 1.00 | - | 0.15 | 72.00 | 70.42-73.58 | - |
| | | CT | 0.80 | 0.56-0.14 | | 0.72 | 0.45-1.13 | | 72.00 | 69.83-74.17 | 0.40 |
| | Log-additive | - | 0.91 | 0.68-1.21 | 0.50 | 0.88 | 0.61-1.27 | 0.50 | - | | |

OR: odds ratio; aOR: odds ratio adjusted for age and gender; CI: confidence interval.

Values in **bold** are statistically significant (p < 0.05).

**Table S2 (cont.).** Genotype risk estimates for the involvement of *PTGS2*, *ABCC4*, *HPGD*, and *SLCO2A1* genetic variants in gastric cancer onset and estimated age at diagnosis.

| SNP | Model | Genotype | Univariate analysis | | | Multivariate analysis* | | | Age at diagnosis | | |
|---|---|---|---|---|---|---|---|---|---|---|---|
| | | | OR | 95% CI | p value | aOR | 95% CI | p value | Median (years) | 95% CI | p value |
| rs4771912 | Codominant | AA | 1.00 | - | 0.52 | 1.00 | - | 0.20 | 72.00 | 70.79-73.21 | - |
| | | AG | 0.84 | 0.57-1.24 | | 0.64 | 0.38-1.08 | | 72.00 | 69.45-74.55 | 0.19 |
| | | GG | 0.61 | 0.17-2.26 | | 0.58 | 0.14-2.46 | | 70.00 | - | 0.71 |
| | Dominant | AA | 1.00 | - | 0.30 | 1.00 | - | 0.071 | 72.00 | 70.79-73.21 | - |
| | | AG-GG | 0.82 | 0.56-1.20 | | 0.64 | 0.39-1.05 | | 72.00 | 70.49-73.51 | 0.24 |
| | Recessive | AA-AG | 1.00 | - | 0.48 | 1.00 | - | 0.53 | 72.00 | 70.87-73.13 | - |
| | | GG | 0.64 | 0.17-2.34 | | 0.64 | 0.15-2.70 | | 70.00 | - | 0.58 |
| | Overdominant | AA-GG | 1.00 | - | 0.40 | 1.00 | - | 0.10 | 72.00 | 70.28-73.72 | - |
| | | AG | 0.85 | 0.57-1.25 | | 0.65 | 0.39-1.09 | | 72.00 | 69.45-74.55 | 0.18 |
| | Log-additive | - | 0.82 | 0.58-1.16 | 0.26 | 0.68 | 0.44-1.05 | 0.077 | - | | |
| rs6492763 | Codominant | TT | 1.00 | - | 0.23 | 1.00 | - | 0.26 | 72.00 | 70.55-73.45 | - |
| | | TC | 0.75 | 0.53-1.06 | | 0.79 | 0.51-1.24 | | 72.00 | 69.89-74.11 | 0.66 |
| | | CC | 0.74 | 0.45-1.23 | | 0.60 | 0.31-1.13 | | 72.00 | 64.37-79.63 | 0.88 |
| | Dominant | TT | 1.00 | - | 0.084 | 1.00 | - | 0.17 | 72.00 | 70.55-73.45 | - |
| | | TC-CC | 0.75 | 0.54-1.04 | | 0.74 | 0.49-1.13 | | 72.00 | 69.94-74.06 | 0.73 |
| | Recessive | TT-TC | 1.00 | - | 0.56 | 1.00 | - | 0.19 | 72.00 | 70.61-73.39 | - |
| | | CC | 0.87 | 0.55-1.39 | | 0.68 | 0.37-1.23 | | 72.00 | 64.37-79.63 | 0.71 |
| | Overdominant | TT-CC | 1.00 | - | 0.20 | 1.00 | - | 0.68 | 72.00 | 70.48-73.52 | - |
| | | TC | 0.81 | 0.59-1.12 | | 0.92 | 0.61-1.38 | | 72.00 | 69.89-74.11 | 0.55 |
| | Log-additive | - | 0.83 | 0.66-1.05 | 0.12 | 0.78 | 0.58-1.05 | 0.10 | - | | |
| rs7993878 | Codominant | GG | 1.00 | - | 0.40 | 1.00 | - | 0.18 | 72.00 | 70.54-73.46 | - |
| | | GA | 0.84 | 0.57-1.25 | | 0.67 | 0.40-1.13 | | 76.00 | 73.59-78.42 | 0.29 |
| | | AA | 0.51 | 0.14-1.83 | | 0.42 | 0.09-1.97 | | 68.00 | 51.28-84.72 | 0.84 |
| | Dominant | GG | 1.00 | - | 0.26 | 1.00 | - | 0.079 | 72.00 | 70.54-73.46 | - |
| | | GA-AA | 0.81 | 0.55-1.18 | | 0.64 | 0.39-1.06 | | 76.00 | 73.52-78.48 | 0.29 |
| | Recessive | GG-GA | 1.00 | - | 0.30 | 1.00 | - | 0.29 | 72.00 | 70.63-73.37 | - |
| | | AA | 0.53 | 0.15-1.90 | | 0.46 | 0.10-2.14 | | 68.00 | 51.28-84.72 | 0.90 |
| | Overdominant | GG-AA | 1.00 | - | 0.42 | 1.00 | - | 0.15 | 72.00 | 70.53-73.47 | - |
| | | GA | 0.85 | 0.57-1.26 | | 0.69 | 0.41-1.15 | | 76.00 | 73.59-78.42 | 0.30 |
| | Log-additive | - | 0.80 | 0.57-1.13 | 0.20 | 0.66 | 0.42-1.04 | 0.064 | | - | |

OR: odds ratio; aOR: odds ratio adjusted for age and gender; CI: confidence interval.
Values in **bold** are statistically significant ($p < 0.05$).

**Table S2 (cont.).** Genotype risk estimates for the involvement of *PTGS2*, *ABCC4*, *HPGD*, and *SLCO2A1* genetic variants in gastric cancer onset and estimated age at diagnosis.

| SNP | Model | Genotype | Univariate analysis | | | Multivariate analysis* | | | Age at diagnosis | | |
|---|---|---|---|---|---|---|---|---|---|---|---|
| | | | OR | 95% CI | p value | aOR | 95% CI | p value | Median (years) | 95% CI | p value |
| rs8002180 | Codominant | TT | 1.00 | - | 0.79 | 1.00 | - | 0.83 | 72.00 | 70.70-73.30 | - |
| | | TC | 1.10 | 0.79-1.53 | | 0.92 | 0.60-1.41 | | 72.00 | 70.27-73.73 | 0.31 |
| | | CC | 0.92 | 0.51-1.67 | | 0.80 | 0.37-1.74 | | 79.00 | 72.08-85.92 | 0.53 |
| | Dominant | TT | 1.00 | - | 0.70 | 1.00 | - | 0.60 | 72.00 | 70.70-73.30 | - |
| | | TC-CC | 1.07 | 0.77-1.47 | | 0.90 | 0.59-1.35 | | 72.00 | 69.90-74.10 | 0.52 |
| | Recessive | TT-TC | 1.00 | - | 0.68 | 1.00 | - | 0.64 | 72.00 | 70.67-73.33 | - |
| | | CC | 0.89 | 0.50-1.58 | | 0.84 | 0.40-1.77 | | 79.00 | 72.08-85.92 | 0.34 |
| | Overdominant | TT-CC | 1.00 | - | 0.53 | 1.00 | - | 0.79 | 72.00 | 70.62-73.38 | - |
| | | TC | 1.11 | 0.80-1.54 | | 0.95 | 0.62-1.43 | | 72.00 | 70.27-73.73 | 0.21 |
| | Log-additive | - | 1.02 | 0.79-1.30 | 0.90 | 0.90 | 0.66-1.25 | 0.54 | - | | |
| rs869951 | Codominant | GG | 1.00 | - | 0.33 | 1.00 | - | 0.43 | 72.00 | 69.52-74.48 | - |
| | | GC | 1.28 | 0.90-1.84 | | 1.35 | 0.85-2.13 | | 72.00 | 70.61-73.39 | 0.89 |
| | | CC | 1.03 | 0.63-1.68 | | 1.28 | 0.69-2.38 | | 71.00 | 69.51-72.50 | 0.71 |
| | Dominant | GG | 1.00 | - | 0.26 | 1.00 | - | 0.20 | 72.00 | 69.52-74.48 | - |
| | | GC-CC | 1.22 | 0.87-1.71 | | 1.33 | 0.86-2.06 | | 72.00 | 70.34-73.66 | 0.79 |
| | Recessive | GG-GC | 1.00 | - | 0.58 | 1.00 | - | 0.79 | 72.00 | 70.78-73.22 | - |
| | | CC | 0.88 | 0.57-1.37 | | 1.08 | 0.62-1.87 | | 71.00 | 69.51-72.50 | 0.64 |
| | Overdominant | GG-CC | 1.00 | - | 0.14 | 1.00 | - | 0.30 | 72.00 | 70.39-73.61 | - |
| | | GC | 1.27 | 0.92-1.75 | | 1.24 | 0.82-1.87 | | 72.00 | 70.61-73.39 | 0.94 |
| | Log-additive | - | 1.06 | 0.84-1.33 | 0.63 | 1.17 | 0.87-1.57 | 0.30 | - | | |
| rs9524821 | Codominant | GG | 1.00 | - | 0.15 | 1.00 | - | 0.33 | 72.00 | 70.08-73.92 | - |
| | | GA | 1.05 | 0.74-1.49 | | 1.22 | 0.78-1.92 | | 72.00 | 70.15-73.85 | 0.48 |
| | | AA | 1.56 | 0.98-2.49 | | 1.57 | 0.85-2.89 | | 71.00 | 67.01-74.99 | 0.86 |
| | Dominant | GG | 1.00 | - | 0.35 | 1.00 | - | 0.21 | 72.00 | 70.08-73.92 | - |
| | | GA-AA | 1.17 | 0.84-1.62 | | 1.31 | 0.86-1.99 | | 72.00 | 70.29-73.71 | 0.63 |
| | Recessive | GG-GA | 1.00 | - | 0.056 | 1.00 | - | 0.23 | 72.00 | 70.88-73.13 | - |
| | | AA | 1.53 | 0.99-2.34 | | 1.42 | 0.81-2.48 | | 71.00 | 67.01-74.99 | 0.62 |
| | Overdominant | GG-AA | 1.00 | - | 0.64 | 1.00 | - | 0.71 | 72.00 | 69.99-74.01 | - |
| | | GA | 0.93 | 0.67-1.28 | | 1.08 | 0.72-1.63 | | 72.00 | 70.15-73.85 | 0.37 |
| | Log-additive | - | 1.21 | 0.96-1.51 | 0.10 | 1.25 | 0.93-1.67 | 0.14 | - | | |

OR: odds ratio; aOR: odds ratio adjusted for age and gender; CI: confidence interval.
Values in **bold** are statistically significant ($p < 0.05$).

**Table S2 (cont.).** Genotype risk estimates for the involvement of *PTGS2*, *ABCC4*, *HPGD*, and *SLCO2A1* genetic variants in gastric cancer onset and estimated age at diagnosis.

| SNP | Model | Genotype | Univariate analysis | | | Multivariate analysis* | | | Age at diagnosis | | |
|---|---|---|---|---|---|---|---|---|---|---|---|
| | | | OR | 95% CI | p value | aOR | 95% CI | p value | Median (years) | 95% CI | p value |
| ***HPGD*** | | | | | | | | | | | |
| rs2612656 | Codominant | AA | 1.00 | - | 0.82 | 1.00 | - | 0.67 | 73.00 | 71.24-74.76 | - |
| | | AG | 1.02 | 0.71-1.48 | | 1.21 | 0.75-1.95 | | 73.00 | 71.92-74.08 | 0.75 |
| | | GG | 0.75 | 0.29-1.94 | | 1.37 | 0.44-4.28 | | 67.00 | 59.07-74.93 | 0.22 |
| | Dominant | AA | 1.00 | - | 0.97 | 1.00 | - | 0.39 | 73.00 | 71.24-74.76 | - |
| | | AG-GG | 0.99 | 0.70-1.41 | | 1.23 | 0.77-1.94 | | 73.00 | 71.50-74.50 | 0.57 |
| | Recessive | AA-AG | 1.00 | - | 0.54 | 1.00 | - | 0.66 | 73.00 | 71.57-74.43 | - |
| | | GG | 0.75 | 0.29-1.92 | | 1.29 | 0.42-3.99 | | 67.00 | 59.07-74.93 | 0.22 |
| | Overdominant | AA-GG | 1.00 | - | 0.84 | 1.00 | - | 0.47 | 73.00 | 71.32-74.68 | - |
| | | AG | 1.04 | 0.72-1.49 | | 1.19 | 0.74-1.91 | | 73.00 | 71.92-74.08 | 0.84 |
| | Log-additive | - | 0.96 | 0.71-1.30 | 0.81 | 1.19 | 0.81-1.76 | 0.37 | - | | |
| rs2555639 | Codominant | TT | 1.00 | - | 0.23 | 1.00 | - | 0.42 | 73.00 | 70.49-75.51 | - |
| | | TC | 0.97 | 0.68-1.39 | | 1.08 | 0.68-1.73 | | 73.00 | 71.28-74.72 | 0.75 |
| | | CC | 1.52 | 0.91-2.55 | | 1.55 | 0.81-2.96 | | 72.00 | 67.67-76.33 | 0.54 |
| | Dominant | TT | 1.00 | - | 0.67 | 1.00 | - | 0.44 | 73.00 | 70.49-75.51 | - |
| | | TC-CC | 1.08 | 0.77-1.50 | | 1.19 | 0.77-1.83 | | 73.00 | 71.26-74.74 | 0.63 |
| | Recessive | TT-TC | 1.00 | - | 0.086 | 1.00 | - | 0.20 | 73.00 | 71.47-74.53 | - |
| | | CC | 1.54 | 0.95-2.52 | | 1.49 | 0.81-2.74 | | 72.00 | 67.67-76.33 | 0.61 |
| | Overdominant | TT-CC | 1.00 | - | 0.47 | 1.00 | - | 0.92 | 73.00 | 71.15-74.85 | - |
| | | TC | 0.88 | 0.63-1.24 | | 0.98 | 0.63-1.51 | | 73.00 | 71.28-74.72 | 0.90 |
| | Log-additive | - | 1.15 | 0.90-1.47 | 0.25 | 1.20 | 0.89-1.63 | 0.24 | - | | |
| rs12500316 | Codominant | CC | 1.00 | - | 0.18 | 1.00 | - | 0.51 | 72.00 | 70.65-73.35 | - |
| | | CT | 0.74 | 0.53-1.04 | | 0.78 | 0.51-1.21 | | 72.00 | 68.82-75.18 | 0.47 |
| | | TT | 1.12 | 0.57-2.18 | | 1.06 | 0.44-2.52 | | 72.00 | 69.44-74.56 | 0.44 |
| | Dominant | CC | 1.00 | - | 0.15 | 1.00 | - | 0.34 | 72.00 | 70.65-73.35 | - |
| | | CT-TT | 0.79 | 0.57-1.09 | | 0.82 | 0.54-1.23 | | 72.00 | 70.01-73.99 | 0.35 |
| | Recessive | CC-CT | 1.00 | - | 0.51 | 1.00 | - | 0.72 | 72.00 | 70.51-73.49 | - |
| | | TT | 1.25 | 0.65-2.41 | | 1.17 | 0.50-2.74 | | 72.00 | 69.44-74.56 | 0.51 |
| | Overdominant | CC-TT | 1.00 | - | 0.069 | 1.00 | - | 0.25 | 72.00 | 70.81-73.19 | - |
| | | CT | 0.73 | 0.53-1.03 | | 0.78 | 0.51-1.19 | | 72.00 | 68.82-75.18 | 0.53 |
| | Log-additive | - | 0.88 | 0.68-1.15 | 0.35 | 0.89 | 0.64-1.25 | 0.52 | - | | |

OR: odds ratio; aOR: odds ratio adjusted for age and gender; CI: confidence interval.

Values in **bold** are statistically significant ($p < 0.05$).

**Table S2 (cont.).** Genotype risk estimates for the involvement of *PTGS2*, *ABCC4*, *HPGD*, and *SLCO2A1* genetic variants in gastric cancer onset and estimated age at diagnosis.

| SNP | Model | Genotype | Univariate analysis | | | Multivariate analysis* | | | Age at diagnosis | | |
|---|---|---|---|---|---|---|---|---|---|---|---|
| | | | OR | 95% CI | p value | aOR | 95% CI | p value | Median (years) | 95% CI | p value |
| rs1346271 | Codominant | GG | 1.00 | - | **0.035** | 1.00 | - | 0.30 | 73.00 | 70.85-75.15 | - |
| | | GC | **0.68** | **0.48-0.96** | | 0.71 | 0.45-1.11 | | 72.00 | 70.06-73.94 | 0.48 |
| | | CC | 1.12 | 0.69-1.82 | | 0.93 | 0.50-1.74 | | 71.00 | 68.85-73.15 | 0.77 |
| | Dominant | GG | 1.00 | - | 0.11 | 1.00 | - | 0.20 | 73.00 | 70.85-75.15 | - |
| | | GC-CC | 0.77 | 0.55-1.06 | | 0.76 | 0.50-1.15 | | 72.00 | 70.63-73.37 | 0.54 |
| | Recessive | GG-GC | 1.00 | - | 0.16 | 1.00 | - | 0.69 | 72.00 | 70.83-73.17 | - |
| | | CC | 1.38 | 0.88-2.17 | | 1.12 | 0.63-2.01 | | 71.00 | 68.85-73.15 | 0.92 |
| | Overdominant | GG-CC | 1.00 | - | **0.011** | 1.00 | - | 0.13 | 72.00 | 70.32-73.68 | - |
| | | GC | **0.66** | **0.48-0.91** | | 0.73 | 0.48-1.10 | | 72.00 | 70.06-73.94 | 0.50 |
| | Log-additive | - | 0.95 | 0.75-1.20 | 0.66 | 0.90 | 0.66-1.21 | 0.48 | - | | |
| rs1426945 | Codominant | GG | 1.00 | - | 0.32 | 1.00 | - | 0.59 | 72.00 | 69.94-74.06 | - |
| | | GA | 0.82 | 0.57-1.17 | | 0.97 | 0.62-1.54 | | 72.00 | 69.43-74.57 | 0.89 |
| | | AA | 1.11 | 0.70-1.75 | | 1.30 | 0.72-2.35 | | 72.00 | 69.41-74.59 | 0.40 |
| | Dominant | GG | 1.00 | - | 0.49 | 1.00 | - | 0.80 | 72.00 | 69.94-74.06 | - |
| | | GA-AA | 0.89 | 0.64-1.24 | | 1.06 | 0.69-1.62 | | 72.00 | 70.15-73.85 | 0.68 |
| | Recessive | GG-GA | 1.00 | - | 0.31 | 1.00 | - | 0.31 | 72.00 | 70.38-73.62 | - |
| | | AA | 1.24 | 0.82-1.88 | | 1.32 | 0.78-2.25 | | 72.00 | 69.41-74.59 | 0.44 |
| | Overdominant | GG-AA | 1.00 | - | 0.15 | 1.00 | - | 0.59 | 72.00 | 70.38-73.62 | - |
| | | GA | 0.79 | 0.57-1.09 | | 0.89 | 0.59-1.35 | | 72.00 | 69.43-74.57 | 0.85 |
| | Log-additive | - | 1.01 | 0.81-1.27 | 0.93 | 1.11 | 0.83-1.49 | 0.47 | - | | |
| rs1863642 | Codominant | GG | 1.00 | - | 0.18 | 1.00 | - | 0.56 | 73.00 | 71.38-74.62 | - |
| | | GT | 0.74 | 0.53-1.03 | | 0.87 | 0.57-1.34 | | 72.00 | 69.75-74.25 | 0.15 |
| | | TT | 1.03 | 0.57-1.85 | | 1.31 | 0.62-2.77 | | 70.00 | 67.19-72.81 | 0.10 |
| | Dominant | GG | 1.00 | - | 0.13 | 1.00 | - | 0.74 | 73.00 | 71.38-74.62 | - |
| | | GT-TT | 0.78 | 0.57-1.08 | | 0.93 | 0.62-1.40 | | 72.00 | 70.22-73.78 | 0.07 |
| | Recessive | GG-GT | 1.00 | - | 0.58 | 1.00 | - | 0.38 | 72.00 | 70.74-73.26 | - |
| | | TT | 1.17 | 0.67-2.08 | | 1.39 | 0.67-2.87 | | 70.00 | 67.19-72.81 | 0.16 |
| | Overdominant | GG-TT | 1.00 | - | 0.064 | 1.00 | - | 0.40 | 72.00 | 70.68-73.32 | - |
| | | GT | 0.74 | 0.53-1.02 | | 0.84 | 0.55-1.27 | | 72.00 | 69.75-74.25 | 0.25 |
| | Log-additive | - | 0.88 | 0.69-1.14 | 0.34 | 1.02 | 0.74-1.41 | 0.90 | - | | |

OR: odds ratio; aOR: odds ratio adjusted for age and gender; CI: confidence interval.
Values in **bold** are statistically significant ($p < 0.05$).

**Table S2 (cont.).** Genotype risk estimates for the involvement of *PTGS2*, *ABCC4*, *HPGD*, and *SLCO2A1* genetic variants in gastric cancer onset and estimated age at diagnosis.

| SNP | Model | Genotype | Univariate analysis | | | Multivariate analysis* | | | Age at diagnosis | | |
|---|---|---|---|---|---|---|---|---|---|---|---|
| | | | OR | 95% CI | p value | aOR | 95% CI | p value | Median (years) | 95% CI | p value |
| rs2303520 | Codominant | GG | 1.00 | - | **0.037** | 1.00 | - | 0.065 | 72.00 | 70.89-73.11 | - |
| | | GA | **1.48** | **1.04-2.09** | | **1.61** | **1.02-2.54** | | 72.00 | 69.12-74.88 | 0.83 |
| | | AA | 0.51 | 0.14-1.79 | | 0.51 | 0.11-2.34 | | 69.00 | 64.20-73.80 | 0.61 |
| | Dominant | GG | 1.00 | - | 0.066 | 1.00 | - | 0.086 | 72.00 | 70.89-73.11 | - |
| | | GA-AA | 1.38 | 0.98-1.93 | | 1.47 | 0.95-2.29 | | 72.00 | 69.05-74.96 | 0.92 |
| | Recessive | GG-GA | 1.00 | - | 0.18 | 1.00 | - | 0.26 | 72.00 | 70.63-73.37 | - |
| | | AA | 0.45 | 0.13-1.59 | | 0.45 | 0.10-2.04 | | 69.00 | 64.20-73.80 | 0.61 |
| | Overdominant | GG-AA | 1.00 | - | **0.021** | 1.00 | - | **0.031** | 72.00 | 70.87-73.13 | - |
| | | GA | **1.51** | **1.07-2.13** | | **1.65** | **1.05-2.59** | | 72.00 | 69.12-74.88 | 0.81 |
| | Log-additive | - | 1.21 | 0.90-1.64 | 0.21 | 1.26 | 0.86-1.84 | 0.24 | - | | |
| rs2555632 | Codominant | TT | 1.00 | - | 0.45 | 1.00 | - | 0.16 | 73.00 | 71.62-74.38 | - |
| | | TC | 1.00 | 0.71-1.41 | | 1.29 | 0.83-2.00 | | 72.00 | 69.22-74.78 | 0.58 |
| | | CC | 1.57 | 0.78-3.14 | | 2.19 | 0.90-5.30 | | **70.00** | **66.67-73.33** | **0.019** |
| | Dominant | TT | 1.00 | - | 0.71 | 1.00 | - | 0.13 | 73.00 | 71.62-74.38 | - |
| | | TC-CC | 1.06 | 0.77-1.47 | | 1.39 | 0.91-2.10 | | 71.00 | 68.72-73.28 | 0.26 |
| | Recessive | TT-TC | 1.00 | - | 0.20 | 1.00 | - | 0.12 | 72.00 | 70.73-73.27 | - |
| | | CC | 1.57 | 0.79-3.10 | | 1.99 | 0.84-4.73 | | **70.00** | **66.67-73.33** | **0.027** |
| | Overdominant | TT-CC | 1.00 | - | 0.83 | 1.00 | - | 0.41 | 72.00 | 70.61-73.39 | - |
| | | TC | 0.96 | 0.69-1.34 | | 1.20 | 0.78-1.85 | | 72.00 | 69.22-74.78 | 0.82 |
| | Log-additive | - | 1.11 | 0.85-1.45 | 0.43 | 1.38 | 0.98-1.94 | 0.066 | - | | |
| rs8752 | Codominant | AA | 1.00 | - | 0.32 | 1.00 | - | 0.30 | 72.00 | 70.13-73.87 | - |
| | | AG | 1.28 | 0.90-1.80 | | 1.39 | 0.90-2.17 | | 72.00 | 70.32-73.68 | 0.60 |
| | | GG | 0.99 | 0.59-1.68 | | 1.39 | 0.71-2.72 | | 72.00 | 66.96-77.04 | 0.31 |
| | Dominant | AA | 1.00 | - | 0.25 | 1.00 | - | 0.12 | 72.00 | 70.13-73.87 | - |
| | | AG-GG | 1.21 | 0.87-1.68 | | 1.39 | 0.91-2.12 | | 72.00 | 70.45-73.55 | 0.46 |
| | Recessive | AA-AG | 1.00 | - | 0.56 | 1.00 | - | 0.64 | 72.00 | 70.53-73.47 | - |
| | | GG | 0.87 | 0.53-1.41 | | 1.16 | 0.62-2.16 | | 72.00 | 66.96-77.04 | 0.41 |
| | Overdominant | AA-GG | 1.00 | - | 0.13 | 1.00 | - | 0.22 | 72.00 | 70.42-73.58 | - |
| | | AG | 1.28 | 0.93-1.76 | | 1.29 | 0.86-1.94 | | 72.00 | 70.32-73.68 | 0.83 |
| | Log-additive | - | 1.07 | 0.84-1.35 | 0.58 | 1.24 | 0.91-1.68 | 0.17 | - | | |

OR: odds ratio; aOR: odds ratio adjusted for age and gender; CI: confidence interval.
Values in **bold** are statistically significant ($p < 0.05$).

**Table S2 (cont.).** Genotype risk estimates for the involvement of *PTGS2*, *ABCC4*, *HPGD*, and *SLCO2A1* genetic variants in gastric cancer onset and estimated age at diagnosis.

| SNP | Model | Genotype | Univariate analysis | | | Multivariate analysis* | | | Age at diagnosis | | |
|---|---|---|---|---|---|---|---|---|---|---|---|
| | | | OR | 95% CI | p value | aOR | 95% CI | p value | Median (years) | 95% CI | p value |
| ***SLCO2A1*** | | | | | | | | | | | |
| rs10935090 | Codominant | CC | 1.00 | - | 0.13 | 1.00 | - | **0.026** | 73.00 | 71.81-74.19 | - |
| | | CT | 1.40 | 0.95-2.06 | | 1.46 | 0.90-2.39 | | **70.00** | **67.56-72.44** | **0.034** |
| | | TT | 2.00 | 0.66-6.04 | | **4.68** | **1.32-16.61** | | **62.00** | **59.61-64.39** | **<0.001** |
| | Dominant | CC | 1.00 | - | 0.054 | 1.00 | - | **0.038** | 73.00 | 71.81-74.19 | - |
| | | CT-TT | 1.44 | 1.00-2.09 | | **1.65** | **1.03-2.63** | | **70.00** | **67.93-72.07** | **0.007** |
| | Recessive | CC-CT | 1.00 | - | 0.028 | 1.00 | - | **0.026** | 72.00 | 70.87-73.14 | - |
| | | TT | 1.86 | 0.62-5.59 | | **4.30** | **1.22-15.16** | | **62.00** | **59.61-64.39** | **<0.001** |
| | Overdominant | CC-TT | 1.00 | - | 0.11 | 1.00 | - | 0.19 | 73.00 | 71.807-74.193 | - |
| | | CT | 1.37 | 0.94-2.02 | | 1.39 | 0.86-2.27 | | 70.00 | 67.56-72.44 | 0.057 |
| | Log-additive | - | **1.40** | **1.01-1.95** | **0.044** | **1.69** | **1.12-2.53** | **0.012** | - | | |
| rs1131598 | Codominant | AA | 1.00 | - | 0.67 | 1.00 | - | 0.59 | 72.00 | 70.55-73.45 | - |
| | | AG | 0.90 | 0.64-1.25 | | 0.81 | 0.52-1.24 | | 73.00 | 71.22-74.78 | 0.86 |
| | | GG | 0.74 | 0.32-1.71 | | 1.07 | 0.40-2.91 | | 69.00 | 62.82-75.18 | 0.54 |
| | Dominant | AA | 1.00 | - | 0.44 | 1.00 | - | 0.38 | 72.00 | 70.55-73.45 | - |
| | | AG-GG | 0.88 | 0.64-1.22 | | 0.83 | 0.55-1.26 | | 72.00 | 70.17-73.84 | 0.96 |
| | Recessive | AA-AG | 1.00 | - | 0.53 | 1.00 | - | 0.76 | 72.00 | 70.88-73.12 | - |
| | | GG | 0.77 | 0.34-1.77 | | 1.17 | 0.44-3.12 | | 69.00 | 62.82-75.18 | 0.46 |
| | Overdominant | AA-GG | 1.00 | - | 0.59 | 1.00 | - | 0.31 | 72.00 | 70.39-73.61 | - |
| | | AG | 0.91 | 0.66-1.27 | | 0.80 | 0.52-1.22 | | 73.00 | 71.22-74.78 | 0.77 |
| | Log-additive | - | 0.88 | 0.67-1.17 | 0.38 | 0.89 | 0.62-1.27 | 0.52 | - | | |
| rs11915399 | Codominant | CC | 1.00 | - | 0.72 | 1.00 | - | 0.12 | 72.00 | 70.27-73.73 | - |
| | | CT | 0.87 | 0.60-1.24 | | **0.61** | **0.38-0.99** | | 73.00 | 71.47-74.53 | 0.13 |
| | | TT | 0.88 | 0.33-2.33 | | 0.75 | 0.22-2.63 | | 74.00 | 59.93-88.07 | 0.52 |
| | Dominant | CC | 1.00 | - | 0.42 | 1.00 | - | **0.043** | 72.00 | 70.27-73.73 | - |
| | | CT-TT | 0.87 | 0.61-1.23 | | **0.62** | **0.39-0.99** | | 73.00 | 71.53-74.47 | 0.11 |
| | Recessive | CC-CT | 1.00 | - | 0.86 | 1.00 | - | 0.81 | 72.00 | 70.64-73.37 | - |
| | | TT | 0.91 | 0.35-2.41 | | 0.86 | 0.25-2.96 | | 74.00 | 59.93-88.07 | 0.59 |
| | Overdominant | CC-TT | 1.00 | - | 0.45 | 1.00 | - | **0.045** | 72.00 | 70.19-73.81 | - |
| | | CT | 0.87 | 0.61-1.25 | | **0.62** | **0.38-1.00** | | 73.00 | 71.47-74.53 | 0.16 |
| | Log-additive | - | 0.89 | 0.65-1.21 | 0.45 | 0.69 | 0.46-1.03 | 0.065 | - | | |

OR: odds ratio; aOR: odds ratio adjusted for age and gender; CI: confidence interval.
Values in **bold** are statistically significant ($p < 0.05$).

**Table S2 (cont.).** Genotype risk estimates for the involvement of *PTGS2*, *ABCC4*, *HPGD*, and *SLCO2A1* genetic variants in gastric cancer onset and estimated age at diagnosis.

| SNP | Model | Genotype | Univariate analysis | | | Multivariate analysis* | | | Age at diagnosis | | |
|---|---|---|---|---|---|---|---|---|---|---|---|
| | | | OR | 95% CI | p value | aOR | 95% CI | p value | Median (years) | 95% CI | p value |
| rs4241362 | Codominant | TT | 1.00 | - | 0.97 | 1.00 | - | 0.49 | 72.00 | 70.65-73.35 | - |
| | | TC | 0.97 | 0.67-1.39 | | 1.13 | 0.71-1.80 | | 73.00 | 68.67-77.63 | 0.82 |
| | | CC | 0.93 | 0.37-2.30 | | 1.86 | 0.65-5.31 | | **65.00** | **62.94-67.06** | **0.019** |
| | Dominant | TT | 1.00 | - | 0.83 | 1.00 | - | 0.42 | 72.00 | 70.65-73.35 | - |
| | | TC-CC | 0.96 | 0.68-1.36 | | 1.20 | 0.77-1.87 | | 72.00 | 68.34-75.67 | 0.82 |
| | Recessive | TT-TC | 1.00 | - | 0.88 | 1.00 | - | 0.29 | 72.00 | 70.87-73.13 | - |
| | | CC | 0.93 | 0.38-2.30 | | 1.79 | 0.63-5.07 | | **65.00** | **62.94-67.06** | **0.024** |
| | Overdominant | TT-CC | 1.00 | - | 0.87 | 1.00 | - | 0.70 | 72.00 | 70.64-73.36 | - |
| | | TC | 0.97 | 0.68-1.39 | | 1.10 | 0.69-1.74 | | 73.00 | 68.67-77.63 | 0.70 |
| | Log-additive | - | 0.97 | 0.72-1.30 | 0.82 | 1.22 | 0.84-1.78 | 0.29 | - | | |
| rs4241365 | Codominant | TT | 1.00 | - | 0.89 | 1.00 | - | 0.53 | 72.00 | 70.71-73.30 | - |
| | | TC | 1.08 | 0.77-1.51 | | 1.10 | 0.71-1.71 | | 72.00 | 69.77-74.23 | 0.58 |
| | | CC | 1.11 | 0.56-2.17 | | 1.65 | 0.68-3.99 | | 74.00 | 67.61-80.39 | 0.61 |
| | Dominant | TT | 1.00 | - | 0.63 | 1.00 | - | 0.47 | 72.00 | 70.71-73.30 | - |
| | | TC-CC | 1.08 | 0.78-1.50 | | 1.17 | 0.77-1.77 | | 72.00 | 69.67-74.33 | 0.71 |
| | Recessive | TT-TC | 1.00 | - | 0.83 | 1.00 | - | 0.30 | 72.00 | 70.65-73.35 | - |
| | | CC | 1.07 | 0.55-2.08 | | 1.59 | 0.67-3.78 | | 74.00 | 67.61-80.39 | 0.56 |
| | Overdominant | TT-CC | 1.00 | - | 0.69 | 1.00 | - | 0.80 | 72.00 | 69.77-74.23 | - |
| | | TC | 1.07 | 0.77-1.49 | | 1.06 | 0.69-1.62 | | 72.00 | 69.77-74.23 | 0.51 |
| | Log-additive | - | 1.07 | 0.82-1.38 | 0.63 | 1.19 | 0.84-1.67 | 0.32 | | - | |
| rs4331673 | Codominant | CC | 1.00 | - | 0.60 | 1.00 | - | 0.19 | 72.00 | 70.80-73.20 | - |
| | | CA | 0.88 | 0.61-1.27 | | 0.92 | 0.58-1.48 | | 72.00 | 68.55-75.45 | 0.48 |
| | | AA | 1.35 | 0.57-3.18 | | 2.61 | 0.92-7.41 | | 65.00 | 62.64-67.36 | 0.32 |
| | Dominant | CC | 1.00 | - | 0.67 | 1.00 | - | 0.84 | 72.00 | 70.80-73.20 | - |
| | | CA-AA | 0.93 | 0.65-1.31 | | 1.05 | 0.67-1.64 | | 72.00 | 68.42-75.58 | 0.69 |
| | Recessive | CC-CA | 1.00 | - | 0.45 | 1.00 | - | 0.072 | 72.00 | 70.63-73.37 | - |
| | | AA | 1.39 | 0.59-3.27 | | 2.67 | 0.95-7.52 | | 65.00 | 62.64-67.36 | 0.33 |
| | Overdominant | CC-AA | 1.00 | - | 0.45 | 1.00 | - | 0.59 | 72.00 | 70.53-73.48 | - |
| | | CA | 0.87 | 0.61-1.25 | | 0.88 | 0.55-1.40 | | 72.00 | 68.55-75.45 | 0.44 |
| | Log-additive | - | 0.98 | 0.73-1.32 | 0.91 | 1.17 | 0.80-1.70 | 0.43 | | - | |

OR: odds ratio; aOR: odds ratio adjusted for age and gender; CI: confidence interval.
Values in **bold** are statistically significant ($p < 0.05$).

**Table S2 (cont.).** Genotype risk estimates for the involvement of *PTGS2*, *ABCC4*, *HPGD*, and *SLCO2A1* genetic variants in gastric cancer onset and estimated age at diagnosis.

| SNP | Model | Genotype | Univariate analysis | | | Multivariate analysis* | | | Age at diagnosis | | |
|---|---|---|---|---|---|---|---|---|---|---|---|
| | | | OR | 95% CI | p value | aOR | 95% CI | p value | Median (years) | 95% CI | p value |
| rs4854784 | Codominant | GG | 1.00 | - | 0.58 | 1.00 | - | 0.73 | 72.00 | 70.10-73.90 | - |
| | | GA | 0.98 | 0.70-1.37 | | 1.06 | 0.69 | | 72.00 | 70.64-73.36 | 0.27 |
| | | AA | 0.75 | 0.43-1.31 | | 1.32 | 0.67-2.62 | | 67.00 | 65.21-68.80 | 0.10 |
| | Dominant | GG | 1.00 | - | 0.66 | 1.00 | - | 0.62 | 72.00 | 70.10-73.90 | - |
| | | GA-AA | 0.93 | 0.68-1.28 | | 1.11 | 0.74-1.68 | | 72.00 | 70.26-73.74 | 0.59 |
| | Recessive | GG-GA | 1.00 | - | 0.30 | 1.00 | - | 0.46 | 72.00 | 70.84-73.16 | - |
| | | AA | 0.76 | 0.44-1.30 | | 1.28 | 0.67-2.46 | | 67.00 | 65.21-68.80 | 0.071 |
| | Overdominant | GG-AA | 1.00 | - | 0.85 | 1.00 | - | 0.97 | 72.00 | 69.98-74.02 | - |
| | | GA | 1.03 | 0.75-1.42 | | 1.01 | 0.67-1.53 | | 72.00 | 70.64-73.36 | 0.16 |
| | Log-additive | - | 0.91 | 0.71-1.15 | 0.42 | 1.12 | 0.82-1.52 | 0.47 | - | | |
| rs6439448 | Codominant | CC | 1.00 | - | 0.31 | 1.00 | - | 0.48 | 72.00 | 70.30-73.70 | - |
| | | CA | 1.14 | 0.81-1.61 | | 1.10 | 0.70-1.72 | | 72.00 | 69.71-74.29 | 0.54 |
| | | AA | 0.52 | 0.17-1.56 | | 0.51 | 0.14-1.82 | | 70.00 | 64.09-75.91 | 0.92 |
| | Dominant | CC | 1.00 | - | 0.67 | 1.00 | - | 0.91 | 72.00 | 70.30-73.70 | - |
| | | CA-AA | 1.08 | 0.77-1.50 | | 1.03 | 0.67-1.58 | | 72.00 | 69.99-74.01 | 0.56 |
| | Recessive | CC-CA | 1.00 | - | 0.18 | 1.00 | - | 0.25 | 72.00 | 70.63-73.37 | - |
| | | AA | 0.49 | 0.16-1.49 | | 0.49 | 0.14-1.75 | | 70.00 | 64.09-75.91 | 0.91 |
| | Overdominant | CC-AA | 1.00 | - | 0.36 | 1.00 | - | 0.58 | 72.00 | 70.28-73.73 | - |
| | | CA | 1.17 | 0.83-1.65 | | 1.13 | 0.73-1.76 | | 72.00 | 69.71-74.29 | 0.53 |
| | Log-additive | - | 1.00 | 0.74-1.34 | 0.98 | 0.95 | 0.65-1.38 | 0.79 | - | | |
| rs7340717 | Codominant | GG | 1.00 | - | 0.30 | 1.00 | - | 0.37 | 72.00 | 70.34-73.66 | - |
| | | GT | 1.15 | 0.82-1.62 | | 1.23 | 0.79-1.91 | | 72.00 | 69.69-74.31 | 0.53 |
| | | TT | 0.77 | 0.45-1.32 | | 0.80 | 0.41-1.56 | | 74.00 | 70.51-77.49 | 0.63 |
| | Dominant | GG | 1.00 | - | 0.72 | 1.00 | - | 0.59 | 72.00 | 70.34-73.66 | - |
| | | GT-TT | 1.06 | 0.77-1.47 | | 1.12 | 0.74-1.69 | | 72.00 | 70.18-73.82 | 0.49 |
| | Recessive | GG-GT | 1.00 | - | 0.19 | 1.00 | - | 0.29 | 72.00 | 70.62-73.38 | - |
| | | TT | 0.72 | 0.43-1.19 | | 0.72 | 0.38-1.34 | | 74.00 | 70.51-77.49 | 0.80 |
| | Overdominant | GG-TT | 1.00 | - | 0.22 | 1.00 | - | 0.22 | 72.00 | 70.31-73.70 | - |
| | | GT | 1.22 | 0.89-1.68 | | 1.30 | 0.86-1.96 | | 72.00 | 69.69-74.31 | 0.60 |
| | Log-additive | - | 0.96 | 0.76-1.21 | 0.71 | 0.98 | 0.73-1.32 | 0.90 | - | | |

OR: odds ratio; aOR: odds ratio adjusted for age and gender; CI: confidence interval.
Values in **bold** are statistically significant ($p < 0.05$).

**Table S2 (cont.).** Genotype risk estimates for the involvement of *PTGS2*, *ABCC4*, *HPGD*, and *SLCO2A1* genetic variants in gastric cancer onset and estimated age at diagnosis.

| SNP | Model | Genotype | Univariate analysis | | | Multivariate analysis* | | | Age at diagnosis | | |
|---|---|---|---|---|---|---|---|---|---|---|---|
| | | | OR | 95% CI | p value | aOR | 95% CI | p value | Median (years) | 95% CI | p value |
| rs7616492 | Codominant | GG | 1.00 | - | 0.83 | 1.00 | - | 0.74 | 72.00 | 69.60-74.40 | - |
| | | GA | 1.08 | 0.77-1.52 | | 1.06 | 0.69-1.65 | | 72.00 | 69.76-74.24 | 0.94 |
| | | AA | 1.15 | 0.70-1.89 | | 0.82 | 0.43-1.59 | | 72.00 | 69.58-74.42 | 0.34 |
| | Dominant | GG | 1.00 | - | 0.58 | 1.00 | - | 0.98 | 72.00 | 69.60-74.40 | - |
| | | GA-AA | 1.10 | 0.79-1.52 | | 1.01 | 0.67-1.52 | | 72.00 | 70.73-73.27 | 0.63 |
| | Recessive | GG-GA | 1.00 | - | 0.68 | 1.00 | - | 0.47 | 72.00 | 69.58-74.42 | - |
| | | AA | 1.10 | 0.69-1.75 | | 0.80 | 0.43-1.48 | | 72.00 | 69.58-74.42 | 0.36 |
| | Overdominant | GG-AA | 1.00 | - | 0.79 | 1.00 | - | 0.61 | 72.00 | 70.26-73.74 | - |
| | | GA | 1.05 | 0.76-1.44 | | 1.11 | 0.74-1.68 | | 72.00 | 69.76-74.24 | 0.85 |
| | Log-additive | - | 1.07 | 0.85-1.35 | 0.55 | 0.95 | 0.70-1.28 | 0.74 | - | | |
| rs7625035 | Codominant | AA | 1.00 | - | 0.29 | 1.00 | - | 0.48 | 72.00 | 70.10-73.90 | - |
| | | AG | 0.85 | 0.61-1.20 | | 0.91 | 0.59-1.40 | | 72.00 | 70.08-73.92 | 0.66 |
| | | GG | 1.49 | 0.74-2.98 | | 1.57 | 0.67-3.71 | | 72.00 | 66.87-77.13 | 0.19 |
| | Dominant | AA | 1.00 | - | 0.62 | 1.00 | - | 0.94 | 72.00 | 70.10-73.90 | - |
| | | AG-GG | 0.92 | 0.67-1.27 | | 0.98 | 0.65-1.49 | | 72.00 | 70.40-73.60 | 0.43 |
| | Recessive | AA-AG | 1.00 | - | 0.20 | 1.00 | - | 0.26 | 72.00 | 70.55-73.45 | - |
| | | GG | 1.58 | 0.80-3.12 | | 1.63 | 0.70-3.79 | | 72.00 | 66.87-77.13 | 0.20 |
| | Overdominant | AA-GG | 1.00 | - | 0.26 | 1.00 | - | 0.52 | 72.00 | 70.08-73.92 | - |
| | | AG | 0.83 | 0.59-1.16 | | 0.87 | 0.57-1.33 | | 72.00 | 70.08-73.92 | 0.83 |
| | Log-additive | - | 1.01 | 0.77-1.32 | 0.94 | 1.07 | 0.76-1.49 | 0.70 | - | | |
| rs7646392 | Codominant | CC | 1.00 | - | 0.63 | 1.00 | - | 0.90 | 72.00 | 69.68-74.32 | - |
| | | CT | 0.87 | 0.61-1.23 | | 0.90 | 0.57-1.41 | | 72.00 | 70.42-73.58 | 0.64 |
| | | TT | 0.82 | 0.51-1.31 | | 0.94 | 0.52-1.71 | | 72.00 | 67.51-76.50 | 0.87 |
| | Dominant | CC | 1.00 | - | 0.35 | 1.00 | - | 0.66 | 72.00 | 69.68-74.32 | - |
| | | CT-TT | 0.86 | 0.62-1.19 | | 0.91 | 0.60-1.38 | | 72.00 | 70.69-73.31 | 0.65 |
| | Recessive | CC-CT | 1.00 | - | 0.57 | 1.00 | - | 1.00 | 72.00 | 70.70-73.30 | - |
| | | TT | 0.88 | 0.57-1.36 | | 1.00 | 0.58-1.73 | | 72.00 | 67.51-76.50 | 0.93 |
| | Overdominant | CC-TT | 1.00 | - | 0.62 | 1.00 | - | 0.67 | 72.00 | 70.08-73.92 | - |
| | | CT | 0.92 | 0.67-1.27 | | 0.91 | 0.60-1.38 | | 72.00 | 70.42-73.58 | 0.61 |
| | Log-additive | - | 0.90 | 0.72-1.13 | 0.35 | 0.96 | 0.72-1.28 | 0.76 | | - | |

OR: odds ratio; aOR: odds ratio adjusted for age and gender; CI: confidence interval.
Values in **bold** are statistically significant ($p < 0.05$).

**Table S2 (cont.).** Genotype risk estimates for the involvement of *PTGS2*, *ABCC4*, *HPGD*, and *SLCO2A1* genetic variants in gastric cancer onset and estimated age at diagnosis.

| SNP | Model | Genotype | Univariate analysis | | | Multivariate analysis* | | | Age at diagnosis | | |
|---|---|---|---|---|---|---|---|---|---|---|---|
| | | | OR | 95% CI | p value | aOR | 95% CI | p value | Median (years) | 95% CI | p value |
| rs9820625 | Codominant | AA | 1.00 | - | 1.00 | 1.00 | - | 0.84 | 72.00 | 69.85-74.15 | - |
| | | AC | 0.99 | 0.68-1.43 | | 1.06 | 0.66-1.71 | | 72.00 | 70.12-73.88 | 0.80 |
| | | CC | 0.99 | 0.63-1.55 | | 1.19 | 0.67-2.09 | | 72.00 | 68.89-75.11 | 0.31 |
| | Dominant | AA | 1.00 | - | 0.94 | 1.00 | - | 0.68 | 72.00 | 69.85-74.15 | - |
| | | AC-CC | 0.99 | 0.70-1.40 | | 1.10 | 0.70-1.72 | | 72.00 | 70.32-73.68 | 0.86 |
| | Recessive | AA-AC | 1.00 | - | 1.00 | 1.00 | - | 0.58 | 72.00 | 70.58-73.42 | - |
| | | CC | 1.00 | 0.68-1.46 | | 1.15 | 0.71-1.87 | | 72.00 | 68.89-75.11 | 0.23 |
| | Overdominant | AA-CC | 1.00 | - | 0.95 | 1.00 | - | 0.94 | 72.00 | 70.19-73.81 | - |
| | | AC | 0.99 | 0.72-1.36 | | 0.98 | 0.64-1.48 | | 72.00 | 70.12-73.88 | 0.43 |
| | Log-additive | - | 0.99 | 0.80-1.24 | 0.96 | 1.09 | 0.82-1.44 | 0.56 | - | | |
| rs9821091 | Codominant | GG | 1.00 | - | 0.32 | 1.00 | - | **0.045** | 72.00 | 69.77-74.23 | - |
| | | GA | 0.86 | 0.61-1.22 | | **0.81** | **0.52-1.28** | | 73.00 | 71.43-74.57 | 0.11 |
| | | AA | 1.22 | 0.76-1.97 | | **1.75** | **0.95-3.20** | | 71.00 | 68.17-73.83 | 0.16 |
| | Dominant | GG | 1.00 | - | 0.70 | 1.00 | - | 0.96 | 72.00 | 69.77-74.23 | - |
| | | GA-AA | 0.94 | 0.68-1.30 | | 0.99 | 0.65-1.50 | | 72.00 | 70.56-73.44 | 0.42 |
| | Recessive | GG-GA | 1.00 | - | 0.21 | 1.00 | - | **0.02** | 72.00 | 70.76-73.24 | - |
| | | AA | 1.33 | 0.86-1.12 | | **1.95** | **1.12-3.40** | | **71.00** | **68.17-73.83** | **0.017** |
| | Overdominant | GG-AA | 1.00 | - | 0.20 | 1.00 | - | 0.085 | 71.00 | 69.06-72.94 | - |
| | | GA | 0.81 | 0.59-1.12 | | 0.70 | 0.46-1.05 | | **73.00** | **71.43-74.57** | **0.018** |
| | Log-additive | - | 1.05 | 0.83-1.32 | 0.70 | 1.19 | 0.89-1.61 | 0.24 | - | | |
| rs9834412 | Codominant | CC | 1.00 | - | 0.94 | 1.00 | - | 0.94 | 73.00 | 71.02-74.98 | - |
| | | CA | 0.94 | 0.67-1.32 | | 1.00 | 0.65-1.55 | | 72.00 | 71.10-72.90 | 0.78 |
| | | AA | 1.02 | 0.53-1.95 | | 1.15 | 0.52-2.55 | | 72.00 | 64.23-79.77 | 0.36 |
| | Dominant | CC | 1.00 | - | 0.78 | 1.00 | - | 0.91 | 73.00 | 71.02-74.98 | - |
| | | CA-AA | 0.96 | 0.69-1.32 | | 1.02 | 0.68-1.55 | | 72.00 | 71.15-72.86 | 0.59 |
| | Recessive | CC-CA | 1.00 | - | 0.90 | 1.00 | - | 0.72 | 72.00 | 70.54-73.46 | - |
| | | AA | 1.04 | 0.55-1.97 | | 1.15 | 0.53-2.50 | | 72.00 | 64.23-79.77 | 0.34 |
| | Overdominant | CC-AA | 1.00 | - | 0.73 | 1.00 | - | 0.94 | 72.00 | 70.22-73.78 | - |
| | | CA | 0.94 | 0.68-1.31 | | 0.98 | 0.64-1.50 | | 72.00 | 71.10-72.90 | 0.92 |
| | Log-additive | - | 0.98 | 0.75-1.27 | 0.86 | 1.04 | 0.75-1.44 | 0.81 | - | | |

OR: odds ratio; aOR: odds ratio adjusted for age and gender; CI: confidence interval.

Values in **bold** are statistically significant ($p < 0.05$)

**Table S3.** Raw quantification data from the real-time polymerase chain reaction.

| | $C_T$ Mean | | | | |
|---|---|---|---|---|---|
| | Target genes | | | | Reference genes |
| | *PTGS2* | *ABCC4* | *HPGD* | *SLCO2A1* | *IPO8* and *HPRT1* |
| GN1 | | 34,44 | 32,02 | 34,58 | 36,17 |
| GN2 | 36,16 | 33,43 | 31,30 | 32,78 | 35,47 |
| GN3 | | 33,91 | 31,44 | 34,81 | 36,34 |
| GN4 | 37,73 | 31,83 | 28,93 | 31,88 | 33,30 |
| GN5 | 37,52 | 33,38 | 29,71 | 33,71 | 34,66 |
| GN6 | 34,11 | 33,70 | 33,41 | 33,93 | 33,57 |
| GN7 | 37,27 | 33,03 | 29,46 | 32,67 | 34,11 |
| GN8 | 34,57 | 31,82 | 28,35 | 31,76 | 33,34 |
| GN9 | | 34,28 | 31,52 | 34,28 | 35,79 |
| GN10 | 36,38 | 32,33 | 29,76 | 32,86 | 33,64 |
| GN11 | 34,17 | 32,46 | 29,46 | 32,23 | 34,11 |
| GN12 | | 33,08 | 29,58 | 32,04 | 33,44 |
| GN13 | 34,70 | 31,97 | 28,45 | 31,96 | 33,06 |
| GN14 | 35,89 | 32,12 | 27,55 | 31,69 | 33,13 |
| GN15 | 35,59 | 32,66 | 30,27 | 32,76 | 33,89 |
| GN16 | 37,76 | 33,41 | 30,45 | 33,83 | 34,29 |
| GN17 | 36,51 | 33,26 | 30,59 | 33,62 | 34,68 |
| GN18 | 34,35 | 31,45 | 27,46 | 31,35 | 32,84 |
| GN19 | 37,31 | 33,62 | 30,85 | 34,01 | 35,25 |
| GN20 | 36,27 | 32,16 | 28,77 | 31,78 | 33,34 |
| GN21 | | 33,14 | 29,86 | 34,14 | 35,45 |
| GN22 | | 34,26 | 30,89 | 34,78 | 37,34 |
| GN23 | | 35,76 | 31,26 | 35,13 | 37,09 |
| GN24 | 35,48 | 32,17 | 28,62 | 31,66 | 34,08 |
| GN25 | 35,34 | 33,41 | 31,19 | 32,98 | 34,95 |
| GN26 | | 34,83 | 30,62 | 34,58 | 35,38 |
| GN27 | | 32,51 | 28,45 | 33,39 | 34,78 |
| GN33 | 35,65 | 32,33 | 29,92 | 32,68 | 34,08 |
| GN34 | 37,78 | 32,71 | 29,38 | 33,42 | 35,06 |
| GN35 | 35,79 | 34,47 | 31,79 | 34,14 | 35,15 |
| GN36 | | 33,13 | 29,69 | 33,03 | 33,89 |
| GN37 | | 33,43 | 29,13 | 33,54 | 34,82 |
| GN38 | 36,67 | 33,41 | 31,10 | 33,44 | 35,51 |
| GN39 | 36,75 | 33,52 | 31,18 | 32,98 | 33,90 |
| GN40 | | 33,73 | 31,02 | 34,46 | 34,36 |
| GN41 | 35,44 | 31,58 | 28,67 | 31,49 | 33,37 |
| GN42 | 37,60 | 33,87 | 30,64 | 34,18 | 36,39 |
| GN43 | 35,80 | 32,13 | 29,97 | 32,75 | 33,12 |
| GN44 | 33,96 | 31,85 | 29,15 | 32,07 | 33,67 |
| GN45 | | 32,58 | 29,54 | 31,76 | 33,06 |
| GN46 | 35,13 | 31,50 | 28,20 | 31,20 | 33,24 |
| GN47 | 36,05 | 31,89 | 28,20 | 32,37 | 33,45 |
| GN49 | 37,90 | 33,29 | 31,60 | 34,39 | 33,00 |
| GN50 | 36,79 | 33,06 | 30,24 | 33,85 | 35,23 |
| GN51 | 35,89 | 33,39 | 29,92 | 33,20 | 34,14 |

$C_T$: cycle threshold.

**Table S3 (cont.).** Raw quantification data from the real-time polymerase chain reaction.

| | $C_T$ Mean | | | | |
|---|---|---|---|---|---|
| | Target genes | | | | Reference genes |
| | *PTGS2* | *ABCC4* | *HPGD* | *SLCO2A1* | *IPO8* and *HPRT1* |
| GN52 | | 35,89 | 33,51 | 36,04 | 37,60 |
| GN53 | 37,21 | 33,85 | 30,99 | 33,93 | 34,70 |
| GN54 | 36,17 | 34,24 | 32,78 | 33,80 | 34,79 |
| GN55 | | 33,39 | 30,24 | 33,38 | 33,86 |
| GN56 | | 33,88 | 31,43 | 34,85 | 36,63 |
| GN57 | 38,28 | 34,12 | 32,36 | 34,84 | 35,95 |
| GN58 | 38,05 | 35,08 | 32,38 | 35,33 | 36,78 |
| GN59 | | 34,17 | 31,20 | 34,61 | 36,00 |
| GN61 | 37,72 | 35,29 | 32,95 | 35,16 | 36,24 |
| GN62 | | 35,40 | 31,76 | | 37,51 |
| GN63 | 37,00 | 36,43 | 33,91 | 35,55 | 36,55 |
| GN64 | 35,45 | 32,28 | 29,66 | 32,17 | 33,86 |
| GN65 | 36,67 | 33,41 | 32,29 | 33,78 | 35,02 |
| GN67 | | 36,15 | 33,15 | 36,80 | 37,91 |
| GN68 | | 34,75 | 33,56 | 34,86 | 36,39 |
| GN69 | | 35,04 | 33,04 | 35,31 | 35,81 |
| GN70 | 36,91 | 34,36 | 32,14 | 34,19 | 35,67 |
| GN71 | 36,80 | 32,48 | 29,89 | 32,86 | 34,17 |
| GN72 | | 32,65 | 30,19 | 32,40 | 34,15 |
| GN73 | | 33,02 | 31,29 | 33,05 | 33,68 |
| GN74 | | 32,75 | 29,32 | 33,21 | 34,67 |
| GN103 | 38,35 | 34,77 | 32,55 | 35,73 | 36,47 |
| GN105 | | 33,89 | 31,23 | 35,32 | 36,12 |
| GN106 | 38,44 | 33,71 | 29,91 | 34,34 | 35,63 |
| GN107 | 37,93 | 33,64 | 31,62 | 34,04 | 35,93 |
| GN108 | | 34,86 | 31,71 | 35,44 | 37,45 |
| GN110 | | 33,98 | 31,80 | 34,88 | 35,31 |
| GN111 | 36,71 | 33,25 | 29,77 | 33,39 | 34,84 |
| GN113 | 37,16 | 33,56 | 31,48 | 33,60 | 35,61 |
| GN114 | | 32,93 | 29,26 | 33,32 | 35,35 |
| GN115 | 38,14 | 34,33 | 31,53 | 33,74 | 35,97 |
| GN119 | | 33,93 | 31,09 | 34,37 | 36,55 |
| GN120 | | 34,19 | 31,39 | 34,77 | 38,03 |
| GN121 | | 34,57 | 33,02 | 36,20 | 37,95 |
| GN122 | | 36,05 | 36,41 | 35,28 | 37,94 |
| GN123 | | 34,43 | 31,37 | 34,08 | 35,01 |
| GN124 | 38,21 | 33,86 | 31,26 | 33,97 | 34,20 |
| GN125 | | 32,38 | 28,52 | 31,65 | 34,24 |
| GN127 | 37,72 | 32,68 | 29,60 | 32,23 | 33,90 |
| GN128 | 38,40 | 34,44 | 32,58 | 35,07 | 36,93 |
| GN129 | | 33,73 | 32,38 | 33,71 | 35,42 |
| GN131 | | 36,05 | 35,16 | 36,85 | 37,70 |
| GN132 | | 37,81 | 35,32 | 33,04 | 37,42 |
| GN133 | | 33,86 | 31,25 | 33,90 | 35,54 |
| GN134 | | 33,58 | 31,02 | 34,30 | 35,46 |

$C_T$: cycle threshold.

**Table S3 (cont.).** Raw quantification data from the real-time polymerase chain reaction.

| | $C_T$ Mean | | | | |
|---|---|---|---|---|---|
| | Target genes | | | | Reference genes |
| | *PTGS2* | *ABCC4* | *HPGD* | *SLCO2A1* | *IPO8* and *HPRT1* |
| GN135 | 38,60 | 33,83 | 30,31 | 33,97 | 35,08 |
| GN136 | | 32,85 | 30,05 | 33,65 | 35,31 |
| GN137 | | 34,01 | 30,44 | 34,16 | 35,49 |
| GN138 | 36,61 | 33,38 | 31,39 | 33,34 | 35,19 |
| GN139 | | 34,42 | 33,30 | 36,01 | 37,30 |
| GN140 | 38,40 | 34,93 | 33,50 | 35,99 | 37,17 |
| GN141 | 37,96 | 34,21 | 32,70 | 35,56 | 36,30 |
| GN143 | | 35,31 | 33,05 | 35,71 | 38,09 |
| GN144 | 37,05 | 34,86 | 32,89 | 35,99 | 37,23 |
| GT1 | 34,99 | 33,61 | 34,29 | 36,01 | 33,72 |
| GT2 | 31,37 | 30,42 | 31,74 | 33,84 | 32,40 |
| GT3 | 35,13 | 32,97 | 32,83 | 34,96 | 33,45 |
| GT4 | 32,40 | 31,71 | 32,22 | 32,59 | 33,15 |
| GT5 | 33,05 | 32,07 | 32,78 | 32,27 | 33,31 |
| GT6 | 34,34 | 33,92 | 30,71 | 33,64 | 34,67 |
| GT7 | 35,13 | 31,44 | 33,70 | 32,88 | 32,86 |
| GT8 | 35,40 | 32,73 | 33,46 | 33,69 | 32,89 |
| GT9 | 37,06 | 33,06 | 31,57 | 34,55 | 35,09 |
| GT10 | 35,28 | 31,40 | 30,90 | 31,66 | 32,66 |
| GT11 | 29,65 | 32,87 | 31,85 | 31,38 | 31,88 |
| GT12 | 30,44 | 31,46 | 30,25 | 31,54 | 31,87 |
| GT13 | 32,21 | 32,29 | 31,56 | 31,01 | 32,76 |
| GT14 | 33,90 | 31,90 | 32,41 | 33,13 | 33,77 |
| GT15 | 32,79 | 31,45 | 32,52 | 31,71 | 32,27 |
| GT16 | 34,52 | 33,13 | 34,07 | 35,00 | 33,56 |
| GT17 | 32,15 | 33,82 | 35,23 | 33,67 | 33,01 |
| GT18 | 31,26 | 31,50 | 32,98 | 31,11 | 32,11 |
| GT19 | 32,46 | 31,45 | 28,87 | 32,47 | 33,64 |
| GT20 | 33,84 | 29,95 | 31,92 | 32,40 | 31,84 |
| GT21 | 32,51 | 32,93 | 29,62 | 33,01 | 32,89 |
| GT22 | 35,20 | 35,22 | 33,80 | | 36,82 |
| GT23 | 36,00 | 32,44 | 32,99 | 35,35 | 34,86 |
| GT24 | | 34,97 | 31,35 | 36,63 | 33,90 |
| GT25 | 35,27 | 31,07 | 29,42 | 33,23 | 32,59 |
| GT26 | 35,04 | 33,25 | 32,26 | 34,11 | 34,21 |
| GT27 | 35,53 | 32,57 | 30,27 | 32,89 | 33,31 |
| GT32 | 33,50 | 32,51 | 30,74 | 34,72 | 33,46 |
| GT33 | | 34,64 | 36,48 | | 39,07 |
| GT34 | | 33,18 | 33,34 | 34,88 | 35,10 |
| GT35 | 34,75 | 34,54 | 35,21 | 36,53 | 35,39 |
| GT36 | 36,35 | 33,98 | 32,59 | 34,91 | 35,89 |
| GT38 | 36,46 | 35,11 | | | 35,87 |
| GT47 | 37,18 | 33,25 | 32,01 | 34,68 | 35,82 |
| GT48 | 35,46 | 34,87 | 34,71 | 38,01 | 36,26 |
| GT51 | 38,80 | 35,57 | 32,17 | | 35,78 |

$C_T$: cycle threshold.

**Table S3 (cont.).** Raw quantification data from the real-time polymerase chain reaction.

| | $C_T$ Mean | | | | |
|---|---|---|---|---|---|
| | Target genes | | | | Reference genes |
| | *PTGS2* | *ABCC4* | *HPGD* | *SLCO2A1* | *IPO8* and *HPRT1* |
| GT52 | 38,10 | 32,97 | 33,77 | 33,09 | 35,79 |
| GT57 | | 36,50 | 35,95 | 35,94 | 35,91 |
| GT62 | | 35,78 | 35,74 | 39,29 | 35,78 |
| GT64 | 35,49 | 32,31 | 35,29 | 33,55 | 34,74 |
| GT65 | | 34,91 | 35,25 | 34,69 | 36,05 |
| GT69 | | 37,76 | | 39,27 | 38,66 |
| GT73 | | 33,05 | 35,90 | 34,75 | 34,66 |
| GT102 | 38,75 | 36,00 | 36,89 | 35,57 | 38,54 |
| GT103 | | 33,54 | | 37,04 | 36,16 |
| GT105 | | 35,07 | 33,95 | 35,74 | 36,81 |
| GT106 | 37,07 | 35,20 | 35,74 | 37,62 | 36,22 |
| GT107 | 35,79 | 34,35 | 34,83 | 36,32 | 36,04 |
| GT108 | | 32,49 | 34,25 | 34,23 | 35,65 |
| GT109 | | 31,24 | 32,86 | 35,04 | 34,60 |
| GT110 | 37,48 | 34,90 | 35,65 | 35,45 | 36,45 |
| GT111 | 35,86 | 32,82 | 33,70 | 36,01 | 35,46 |
| GT113 | 33,45 | 34,01 | 36,50 | 37,20 | 35,40 |
| GT114 | 35,10 | 32,39 | 32,77 | 35,13 | 35,35 |
| GT116 | | 34,02 | 36,18 | 33,82 | 36,19 |
| GT117 | 33,87 | 33,63 | 31,94 | 34,76 | 33,48 |
| GT118 | | 34,92 | 37,05 | | 37,41 |
| GT119 | 37,69 | 36,09 | 34,38 | 38,95 | 35,98 |
| GT120 | 37,84 | 34,04 | | 37,88 | 38,30 |
| GT122 | 35,64 | 33,53 | 36,40 | 34,72 | 34,76 |
| GT123 | 36,76 | 33,81 | 33,90 | 35,22 | 34,21 |
| GT124 | | 33,62 | 33,84 | 34,05 | 34,74 |
| GT128 | | 34,84 | | 37,11 | 37,40 |
| GT129 | 37,43 | 32,77 | 34,23 | 35,01 | 34,79 |
| GT131 | | 34,18 | 35,64 | 35,65 | 37,86 |
| GT132 | | 35,99 | 37,19 | | 37,86 |
| GT133 | | 34,11 | 35,61 | 33,68 | 36,08 |
| GT134 | 35,00 | 30,82 | 34,71 | 34,33 | 33,75 |
| GT135 | 31,82 | 32,42 | 33,58 | 36,87 | 34,09 |
| GT138 | 36,55 | 34,50 | 33,97 | 34,00 | 35,49 |
| GT139 | 36,69 | 34,12 | 36,01 | 35,36 | 36,21 |
| GT141 | | 36,50 | | 38,78 | 37,25 |
| GT142 | 36,47 | 33,40 | 34,06 | 37,79 | 35,33 |
| GT143 | 36,94 | 35,66 | 34,37 | 37,74 | 36,61 |
| GT145 | 37,92 | 37,32 | 35,21 | 37,73 | 36,19 |
| GT147 | | 33,52 | 33,20 | 35,17 | 36,06 |
| GT148 | | 34,55 | 35,07 | 35,93 | 35,54 |
| GT149 | 36,92 | 33,44 | 35,25 | 33,62 | 37,07 |
| GT150 | | 33,65 | | 34,22 | 34,62 |
| GT151 | 35,96 | 33,88 | 34,07 | 36,51 | 35,54 |
| GT152 | 34,70 | 32,72 | 35,85 | 37,07 | 34,18 |

$C_T$: cycle threshold.

**Table S3 (cont.).** Raw quantification data from the real-time polymerase chain reaction.

| | $C_T$ Mean | | | | |
|---|---|---|---|---|---|
| | Target genes | | | | Reference genes |
| | *PTGS2* | *ABCC4* | *HPGD* | *SLCO2A1* | *IPO8* and *HPRT1* |
| GT153 | 35,62 | 33,23 | 33,42 | 34,61 | 35,87 |
| GT154 | | 33,74 | 33,32 | 35,56 | 36,02 |
| GT155 | 37,64 | 32,72 | 33,14 | 33,32 | 35,36 |
| GT156 | 37,31 | 32,91 | 36,33 | 33,88 | 37,14 |
| GT157 | 37,87 | 34,22 | 34,75 | 36,47 | 35,91 |
| GT158 | 38,06 | 34,18 | 35,66 | | 36,89 |
| GT159 | 33,61 | 32,97 | 34,73 | 36,24 | 34,65 |
| GT160 | 37,41 | 33,32 | 33,57 | 34,43 | 34,50 |
| GT162 | | 35,68 | 35,35 | 36,36 | 36,67 |
| GT163 | 34,49 | 35,83 | | 36,31 | 35,87 |
| GT164 | | 36,08 | 37,32 | 36,77 | 37,43 |
| GT165 | 38,32 | 35,89 | 35,26 | | 36,75 |
| GT166 | | 35,41 | 35,87 | 36,33 | 35,94 |
| GT167 | 36,90 | 34,87 | 36,85 | | 37,29 |
| GT168 | | 34,58 | 34,88 | 36,06 | 36,31 |

$C_T$: cycle threshold.
